# A microRNA expression signature in infant t(4;11) KMT2A::AFF1+ BCP‐ALL uncovers novel therapeutic targets

**DOI:** 10.1002/hem3.70353

**Published:** 2026-04-23

**Authors:** Camille Malouf, Alasdair Duguid, Kirsten S. Vrenken, Tom Leah, Ragini Medhi, Giuseppina Camiolo, Leslie Nitsche, Hélène Jakobczyk, Rishi S. Kotecha, Richard A. Anderson, Neil A. Barrett, Owen P. Smith, Ronald W. Stam, Katrin Ottersbach

**Affiliations:** ^1^ Centre for Regenerative Medicine, Institute for Regeneration and Repair University of Edinburgh Edinburgh UK; ^2^ Princess Máxima Center for Pediatric Oncology Utrecht The Netherlands; ^3^ Leukaemia Translational Research Laboratory, Telethon Kids Cancer Centre, Telethon Kids Institute University of Western Australia Perth Western Australia Australia; ^4^ Curtin Medical School Curtin University Perth Western Australia Australia; ^5^ Department of Clinical Haematology, Oncology, Blood and Marrow, Transplantation Perth Children's Hospital Perth Western Australia Australia; ^6^ Centre for Reproductive Health, Institute for Regeneration and Repair University of Edinburgh Edinburgh UK; ^7^ Department of Haematology Children's Health Ireland Dublin Ireland; ^8^ National Children's Cancer Service Children's Health Ireland Dublin Ireland; ^9^ Trinity College Dublin Dublin Ireland

## Abstract

Infants and children with KMT2A::AFF1+ leukemia have a dismal prognosis and are therefore in urgent need for more efficient and less aggressive therapy. In this study, we investigated three microRNAs that are downregulated in KMT2A::AFF1+ B‐cell precursor acute lymphoblastic leukemia (BCP‐ALL): miR‐194, miR‐99b, and miR‐125a‐5p. When overexpressed, all three microRNAs impaired the survival of KMT2A::AFF1+ leukemic blasts and the maintenance of KMT2A::AFF1+ BCP‐ALL. We identified microRNA target genes responsible for this phenotype that are upregulated in KMT2A::AFF1+ BCP‐ALL: CA5B, PPP3CA, and PPP2R5C. Importantly, using a drug‐repurposing approach, we found that inhibition of CA5B, PPP3CA, and PP2A by acetazolamide, tacrolimus, and LB‐100, respectively, showed high toxicity toward KMT2A::AFF1+ leukemic blasts and reduced leukemia burden in vivo. Furthermore, acetazolamide was able to prolong the survival of patient‐derived xenotransplant models in combination with infant ALL induction therapy. This study highlights how the unique microRNA expression signature of patients with KMT2A::AFF1+ BCP‐ALL can be used to uncover novel therapeutic avenues and accelerate drug repurposing. It also indicates potential new drug combinations for less toxic chemotherapy.

## Graphical abstract

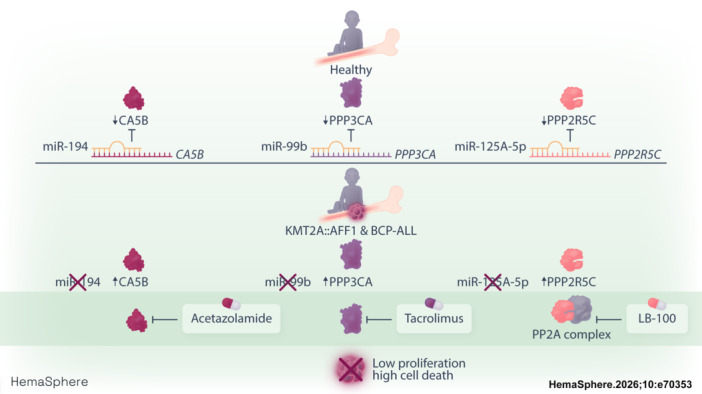

## INTRODUCTION

T(4;11) KMT2A::AFF1+ B‐cell precursor acute lymphoblastic leukemia (BCP‐ALL) is one of the most aggressive malignancies occurring in infants less than 1 year of age.^1^ It is characterized by a poor prognosis and a high frequency of relapse.^2^, ^3^, ^4^ KMT2A::AFF1+ pro‐B leukemic blasts display uncontrolled proliferation and harbor an immature pro‐B lymphoid phenotype (CD19^+^ CD10^−^) that retains stem cell (CD34) and myeloid features (CD33).^5^ Patients with KMT2A::AFF1+ BCP‐ALL are also more prone to lineage switching during the course of their therapy and at relapse, suggesting that the cell of origin has a dual myeloid/lymphoid potential.^6^

Retrospective analysis of neonatal blood spots and studies in monozygotic twins have confirmed the prenatal origin of infant t(4;11) KMT2A::AFF1+ BCP‐ALL.^7^, ^8^ The KMT2A::AFF1 fusion oncogene induces a unique epigenetic signature in leukemic blasts characterized by a distinct histone hypermethylation pattern and a strong upregulation of MLL target genes.^9^, ^10^, ^11^, ^12^ More recent studies have also highlighted the crucial role of the fetal signature and microRNAs in the initiation, maintenance, and lineage plasticity of KMT2A::AFF1‐driven leukemogenesis.^13^, ^14^, ^15^, ^16^, ^17^, ^18^

MicroRNAs are aberrantly expressed in leukemia and are crucial to the maintenance of the hematopoietic stem cell (HSC) pool and blood differentiation.^19^, ^20^, ^21^, ^22^ In our previous study, we found a low/absent expression of three microRNAs in the leukemic blasts of patients with KMT2A::AFF1+ BCP‐ALL: miR‐194, miR‐99b, and miR‐125a‐5p.^14^ These microRNAs have been studied in hematopoiesis and other subtypes of leukemia,^21^, ^23^ but their specific role in KMT2A::AFF1+ BCP‐ALL remains to be investigated.

In this study, we show that miR‐194, miR‐99b, and miR‐125a‐5p overexpression impaired the proliferation and survival of leukemic cells in vitro, which led to an increased survival of mice with KMT2A::AFF1+ BCP‐ALL. Using published gene expression data sets, we identified three target genes upregulated in patients with KMT2A::AFF1+ BCP‐ALL that recapitulate the microRNA‐mediated phenotype: CA5B (miR‐194 target), PPP3CA (miR‐99b target), and PPP2R5C (miR‐125a‐5p target). Knockout of these genes using CRISPR‐Cas9 in leukemic cells recapitulated the phenotype observed upon their respective microRNA overexpression in all three cases: decreased proliferation and increased apoptosis. Most importantly, there are clinically approved drugs that can inhibit the enzymatic activity of all three genes: acetazolamide (CA5B), tacrolimus (PPP3CA), and LB‐100 (PP2A complex). These inhibitors were detrimental to the proliferation and survival of KMT2A::AFF1+ leukemia cell lines and primary cells derived from three infants with KMT2A::AFF1+ BCP‐ALL, and all three drugs decreased KMT2A::AFF1+ leukemia burden in vivo. Importantly, acetazolamide was also able to impair KMT2A::AFF1+ BCP‐ALL progression in combination with the current treatment regimen for infant BCP‐ALL. Overall, this study identified three novel tumor suppressor microRNAs in KMT2A::AFF1‐driven leukemogenesis (miR‐194, miR‐99b, and miR‐125a‐5p) that can negatively affect the expression of three oncogenes (CA5B, PPP3CA, and PPP2R5C). This led to the identification of novel therapeutic avenues that could be used in the clinic to improve current therapeutic regimens. Hence, the microRNA expression signature of KMT2A::AFF1+ BCP‐ALL patients can be used to accelerate the drug discovery process for infant KMT2A::AFF1+ BCP‐ALL.

## METHOD

### MicroRNA profiling of patients and primers

Primary patient samples and data (provided in Supporting Information S12: Table 5) used in this study were provided by VIVO Biobank, supported by Cancer Research UK & Blood Cancer UK (Grant no. CRCPSC‐Dec21\100003). The data were taken from our previous study (Malouf et al.,^14^ PMID: 34111240). All primers used in this study are listed in Supporting Information S13: Table 6.

### Identification of differentially methylated regions

Differentially methylated regions (DMRs) in leukemic blasts from KMT2A::AFF1+ infant B‐ALLs (*n* = 2), and healthy B‐cell progenitors (hBCP; *n* = 2) were obtained from publicly available data.^24^ Identified DMRs in the comparison KMT2A::AFF1+ infant B‐ALLs (AFF1r) versus hBCP were processed using the GenomicRanges package (version 1.58.0^25^). IGV^26^ was used for visualization with human genome assembly GRCh37 as the reference and promoter annotations from Eukaryotic Promoter Database as the EPDnew Promoters track.^27^

### Sorting of human fetal liver hematopoietic cells

Anonymized human fetal livers (FLs) from morphologically normal 10–20‐week‐old fetuses were collected following elective medical termination of pregnancy at the Royal Infirmary of Edinburgh after informed written consent (approved by the Lothian Research Ethics Committee, Reference: 08/S1101/1). FLs were dissociated in Flow Cytometry Staining Buffer (ThermoFisher Cat# 00‐4222‐26) using a 21G × 15 mm needle attached to a syringe (BD Microlance Cat# 10472204‐X and 3000185) and further CD34‐enriched using the CD34 MicroBead Kit Ultrapure human (Millitenyi Cat# 130‐100‐453) according to the manufacturer's instructions. The CD34^+^ fraction was stained with the following antibodies (lineage cocktail on APC) in Flow Cytometry Staining Buffer (ThermoFisher Cat# 00‐4222‐26): APC anti‐human CD2 antibody (clone RPA‐2.10, Biolegend Cat# 300213), APC anti‐human CD3 antibody (clone HIT3, Biolegend Cat# 300311), APC anti‐human CD14 antibody (clone M5E2, Biolegend Cat# 301807), APC anti‐human CD16 antibody (clone 3G8, Biolegend Cat# 302011), APC anti‐human CD56 antibody (clone HCD56, Biolegend Cat# 318309), APC anti‐human CD235ab antibody (clone HIR2, Biolegend Cat# 306607), Alexa Fluor® 700 anti‐human CD34 antibody (clone 561, Biolegend Cat# 343621), PE anti‐human CD45RA antibody (clone HI100, Biolegend Cat# 304107), PE/Cy7 anti‐human CD10 antibody (clone eBIOCB‐CALLA, ThermoFisher Cat# 25‐0106‐42), BrilliantViolet™ 421 anti‐human CD90 (clone 5E10, Biolegend Cat# 328121), BrilliantViolet™ 510 anti‐human CD38 (clone HB7, Biolegend Cat# 356633), and BrilliantViolet™ 605 anti‐human CD19 (clone HIB19, Biolegend Cat# 302243). The CD34^−^ fraction was stained with the following antibodies in Flow Cytometry Staining Buffer (ThermoFisher Cat# 00‐4222‐26): Alexa Fluor® 700 anti‐human CD34 antibody (clone 561, Biolegend Cat# 343621), APC anti‐human CD3 antibody (clone HIT3, Biolegend Cat# 300311), FITC anti‐human CD33 antibody (clone P67.6, Biolegend Cat# 366619), PE anti‐human CD56 antibody (clone HCD56, Biolegend Cat# 318305), and PE/Cy7 anti‐human CD20 antibody (clone 2H7, Biolegend Cat# 302311). Cells were incubated on ice for 20 min, washed twice with Flow Cytometry Staining Buffer, and resuspended in diluted SYTOX AADvanced (ThermoFisher Cat# S10274) to exclude dead cells. Cells were sorted on a BD FACSAria™ II (BD Biosciences) into QIAzol lysis reagent (QIAGEN, Cat# 79306) for RNA extraction. We used the following gating strategy for human hematopoietic stem and progenitor cells: HSC (Lin^−^ CD34^+^ CD38^−^ CD19^−^ CD45RA^−^ CD90^+^), MPP (Lin^−^ CD34^+^ CD38^−^ CD19^−^ CD45RA^−^ CD90^−^), LMPP (Lin^−^ CD34^+^ CD38^−^ CD19^−^ CD45RA^+^), pre‐pro‐B (Lin^−^ CD34+ CD38^+^/low CD10^−^ CD19^+^), and pro‐B (Lin^−^ CD34^+^ CD38^+^/low CD10^+^ CD19^+^). We used the following gating strategy for committed and/or mature hematopoietic cells: B‐lymphoid cells (CD34^−^ CD20^+^), NK cells (CD34^−^ CD56^+^), T‐lymphoid cells (CD34^−^ CD3^+^), and myeloid cells (CD34^−^ CD33^+^). Reverse transcription and reverse transcription quantitative polymerase chain reaction (RT‐qPCR) for microRNA were performed using the TaqMan MicroRNA Reverse Transcription Kit and TaqMan Universal Master Mix II, no UNG, according to the manufacturer's instructions (ThermoFisher Cat# 4366596 and #4440047). MicroRNA expression was assessed using individual Taqman assays (ThermoFisher Assay ID 002216, 00456, 000493, 000436, 002198, and 001973).

### Human cord blood and bone marrow sample processing

The human cord blood (CB) was obtained from the Edinburgh Reproductive Tissue Biobank (ERTBB). Blood was diluted 1:1 in PBS, separated with an equal volume of Ficoll (HISTOPAQUE‐1077, Sigma 10771‐500ML), and centrifuged at 1000 × *g* for 30–40 min. The peripheral blood mononuclear cell (PBMC) layer was removed and processed with the CD34 microbead kit, human (Milliteny Biotec, Cat# 130‐046‐702). The human bone marrow (BM) was collected at the IRR and processed similarly.

### Mice

The majority of the animal work was carried out under the regulation of the UK Home Office. Males and females were mated to obtain E14 embryos, with the day of plug detection being counted as Day 0 of embryonic development. The Kmt2a::AFF1 and VEC‐Cre mice were from our previous studies.^15^ The Kmt2a::AFF1 line was obtained from Professor Terry Rabbitts^28^ and the VEC‐Cre line was obtained from Professor Nancy Speck.^29^ The animal experiment, including induction therapy, was carried out according to Dutch legislation and approved by the Animal Ethics Committee (Approval Number: AVD39900202216167), Prinses Máxima Center for Paediatric Oncology, Utrecht, the Netherlands.

### Cell lines

PER‐494 (CVCL_A8JU), RS4;11 (ATCC®CRL‐1873™), SEM (DSMZ ACC 546), Nalm6 (ATCC®CRL‐3273™), MV4;11 (ATCC®CRL‐9591™), MOLM‐13 (DSMZ ACC 554), THP‐1 (ATCC®TIB‐200™), Nomo‐1 (DSMZ ACC 542), and Kasumi1 (ATCC®CRL‐2724™) cells were maintained in 20% fetal calf serum (FCS) 1% P/S 1% l‐glut RPMI (Roswell Park Memorial Institute) 1640 (cells kindly provided by Professor Mark Dawson, Professor Brian Huntly, and Dr. Rishi Sury Kotecha).

### Sorting of E14 FL ckit^+^ CD34^+^ and CD45^+^ CD34^−^ cells

Dissociated FL cells were stained using the following antibody mix in Flow Cytometry Staining Buffer (ThermoFisher Cat# 00‐4222‐26): APC anti‐mouse CD117 (ckit) antibody (clone 2B8, Biolegend Cat# 105811), FITC rat anti‐mouse CD34 (clone RAM34, BD Cat# 560238), and PE CD45 monoclonal antibody eBioscience™ (clone 30‐F11, ThemoFisher Cat# 12‐0451‐82). Cells were incubated with the antibodies for 20 min on ice, washed twice with Flow Cytometry Staining Buffer, and resuspended in diluted SYTOX AADvanced (ThermoFisher Cat# S10274) to exclude dead cells. Cells were sorted on a BD FACSAria™ II (BD Biosciences) into QIAzol lysis reagent (QIAGEN, Cat# 79306) for RNA extraction.

### Sorting of E14 FL E‐SLAM HSC

Lineage depletion was performed on dissociated FL cells using the EasySep™ Mouse Hematopoietic Progenitor Cell Isolation Kit (STEMCELL Technologies Cat# 19856) according to the manufacturer's instructions. Cells were stained using the following antibody mix in Flow Cytometry Staining Buffer (ThermoFisher Cat# 00‐4222‐26): FITC anti‐mouse CD45 antibody (clone 30‐F11, Biolegend Cat# 103017), APC anti‐mouse CD48 antibody (clone HM48‐1, Biolegend Cat# 103411), Pacific Blue™ anti‐mouse CD150 antibody (clone TC15‐12F12.2, Biolegend Cat# 115923), and PE anti‐mouse EPCR monoclonal antibody (clone eBio1560 [1560], ThermoFisher Cat# 12‐2012‐82). Cells were incubated with the antibodies for 20 min on ice, washed twice with Flow Cytometry Staining Buffer, and resuspended in diluted SYTOX AADvanced (ThermoFisher Cat# S10274) to exclude dead cells. Cells were sorted on a BD FACSAria™ II (BD Biosciences) into QIAzol lysis reagent (QIAGEN, Cat# 79306) for RNA extraction.

### Electroporation of SEM cells with mimics and siRNA

Transfection of SEM cells with mimics for miR‐194 and miR‐99, and miR‐125, and the control mimic, was performed in RPMI 1640 20% FCS 1% P/S 1% l‐glut. We used 200 μL of SEM cells at a concentration of 6 × 10^6^ cells/mL and 300 pmole of *mir*Vana® miRNA mimic (Life Technologies Cat# MC10004, MC11021, MC12561) or control (Life Technologies Cat# 4464058). We used the Gene Pulser Xcell Electroporation system (BioRad Cat# 1652660) with GenePulser/MicroPulser Electroporation Cuvette, 0.4 cm gap (BioRad Cat# 1652088). The following electroporation program was used: square wave length, 10 ms, 350 V. After electroporation, cells were allowed to stand for 15 min at room temperature, before being diluted 1:20 in RPMI 1640 20% FCS 1% P/S 1% l‐glut.

### Lentivirus production in 293T cells and transduction experiments

293T cells (ATCC®CRL‐3216™) were maintained in 10% FCS, 1% P/S, and 1% l‐glut Dulbecco's modified Eagle medium (DMEM). Lentiviral vectors for the microRNA overexpression were purchased from System Biosciences: Human pre‐microRNA Expression Construct Lenti‐miR‐194‐1 (Cat# PMIRH1941PA‐1), Human pre‐microRNA Expression Construct Lenti‐miR‐99b (Cat# PMIRH99b‐1), Human pre‐microRNA Expression Construct Lenti‐miR‐125a (Cat# PMIRH1125a‐PA‐1), and empty pMIRH vector (Cat# PMIRH CD511B‐1). For the CRISPR‐Cas9, we used the following vectors to produce two lentiviruses: pKLV2‐U6gRNA5(BbsI)‐sEF1aBFP‐W (AddGene Cat# 67974) and LentiCas9‐EGFP (AddGene Cat# 63592). We used pMD2.G and psPAX2 as packaging vectors (Addgene Cat# 12259 and 12260). The lentiviral vector, and pMD2G and psPAX2 vectors were transfected into HEK293T cells using 1 mg/mL of polyethyleneimine, high molecular weight (Sigma‐Aldrich Cat# 408727), and serum‐free DMEM. The DNA solution was briefly vortexed and allowed to stand at room temperature for 15 min, before addition to a plate of 60%–70% confluent 293T cells. The morning after, transfection media were removed and replaced with fresh media for virus collection at 36–48 h after transfection. The supernatant containing lentiviral particles was filtered before transduction of FL LSK or leukemia cell lines through a Millex‐hV 0.45 µm PVDF 33 mm Gamma Sterilized filter (Millipore Europe Cat# SLHVM33RS). For leukemia cell lines, the supernatant was added directly to cells along with polybrene at a final concentration of 4 μg/mL of polybrene (Santa Cruz Biotechnology Cat# sc‐134220). For mouse FL LSK cells and Kmt2a::AFF1+ pMIRH‐128a pro‐B leukemic blasts, the supernatant (MOI > 10) was added on a non‐treated plate coated with RetroNectin® Recombinant Human Fibronectin Fragment according to the manufacturer's instructions (Takara Bio Inc Cat# T100A). Cells were maintained in StemPro™‐34 SFM (1X) (ThermoFisher Cat# 10639011) supplemented with 100 ng/mL SCF, 100 ng/mL TPO, and 50 ng/mL Flt3 (PeproTech EC Ltd, Cat# 315‐14‐10, 250‐03‐10, and 250‐31L‐10) over a 24‐h period. After 3 days in culture, LSK cells were collected and used for further studies.

### Cell proliferation, apoptosis assay, cell cycle assay, and in vitro drug studies

Cell proliferation assays were conducted in a 6‐well plate. Cells were counted using Trypan Blue Solution (Life Technologies, Cat# 15250061). Apoptotic cells were detected by double‐staining using PE‐AnnexinV (Biolegend Cat# 640907) and SYTOX Blue Dead Cell Stain (ThermoFisher Cat# S34857) for transduced cells with pMIRH. Apoptotic cells were detected by double staining using PE‐AnnexinV (Biolegend Cat# 640907) and SYTOX Red Dead Cell Stain (ThermoFisher Cat# S34859) for transduced cells with pKLV2‐U6gRNA5(BbsI)‐sEF1aBFP‐W (AddGene Cat# 67974) and LentiCas9‐EGFP. Staining was carried out in the AnnexinV Binding Buffer according to the manufacturer's instructions (BD Biosciences Cat# 556454). For the caspase activity assay, we used the CellEvent™ Caspase‐3/7 Green Flow Cytometry Assay Kit (Invitrogen, Cat# C10427) and followed the manufacturer's protocol. For the cell cycle assay, cells were washed with PBS and stained with eBioscience Fixable Viability Dye eFluor780 (Invitrogen, Cat# 65‐0865‐14), then washed and fixed with a BD Cytofix/Cytoperm kit (BD Biosciences 554714) for 20 min, and finally stained with DAPI 1:1000. For drug studies, we used the following drugs reconstituted in DMSO (Acetazolamide and TACROLIMUS) or sterile water (LB‐100): Acetazolamide (Generon Cat# HY‐B0782), TACROLIMUS (Cambridge Bioscience Cat# F001), or LB‐100 (Stratech, Cat# B4846‐APE).

### Transplantation of SEM leukemic cells into NSG mice for the rescue experiment and flow cytometry analysis of recipients

SEM leukemic cells that express pMIRH, pMIRH‐194, pMIRH‐99b, or pMIRH‐125a‐5p were sorted based on GFP expression and tail vein‐injected into non‐irradiated NSG mice (2500 cells/mouse). Mice were killed once leukemia was established, and single‐cell suspensions from peripheral blood and tissues (BM, spleen, and liver) were analyzed by flow cytometry using the following antibodies: APC anti‐human CD45 (clone 2D1, Biolegend Cat# 368511), PE anti‐human CD19 (clone 4G7 Biolegend Cat# 392505), Brilliant Violet 605™ anti‐human CD10 (clone HI10a, Biolegend Cat# 312221), Brilliant Violet 421™ anti‐human CD33 (clone P67.6, Biolegend Cat# 366621), Brilliant Violet 711™ anti‐human IgM (clone MHM‐88, Biolegend Cat# 314539), and PE/Cy7 anti‐mouse CD45 (clone 30‐F11, Biolegend Cat# 103113). Cells were incubated with the antibodies for 20 min on ice, washed twice with Flow Cytometry Staining Buffer, and resuspended in diluted SYTOX AADvanced (ThermoFisher Cat# S10274) to exclude dead cells. Data were acquired on a BD LSRFortessa™ (BD Biosciences).

### Transplantation of FL LSK cells or mouse GFP+ Kmt2a::AFF1+ pMIRH‐128a leukemic cells for the rescue experiment and flow cytometry analysis of recipients

Transplantation recipients (female Ly5.1/2 mice aged 8–12 weeks old) were irradiated with a total dose of 9.2 Gy (2 doses of 4.6 Gy, 3 h apart, with a split adaptor). Transduced GFP+ LSK or Kmt2a::AFF1+ pMIRH‐128a cells were transplanted through a tail vein injection (50,000 LSK cells/mouse or 10,000 Kmt2a::AFF1+ pMIRH‐128a cells/mouse) into irradiated Ly5.1/2 recipients along with 20,000 helper BM cells (Ly5.1/1). Mice were administered antibiotics after transplantation through their drinking water (0.1 mg/mL enrofloxacin and 10% Baytril solution from Bayer) and bled on a monthly basis. Blood counts were measured on a Celltac MEK‐6500K (Nihon Kohden). For flow cytometry analysis of peripheral blood and tissues, red blood cell lysis was carried out with BD Pharm Lyse™ lysing solution according to the manufacturer's instructions (BD Biosciences Cat# 555899). Cells were stained in Flow Cytometry Staining Buffer using the following mixture of antibodies: APC‐eFluor 780‐CD45.2 monoclonal antibody eBioscience™ (clone 104, ThermoFisher Cat# 47‐0454‐82), PE‐CD45.1 monoclonal antibody eBioscience™ (clone A20, ThermoFisher Cat# 12‐0543‐83), eFluor450‐CD11b monoclonal antibody eBioscience™ (clone M1/70, ThermoFisher Cat# 48‐0112‐80), Alexa Fluor® 700 anti‐mouse Ly‐6G/Ly‐6C (Gr‐1) antibody (clone RB6‐8C5, Biolegend Cat# 108422), PE/Cy7 anti‐mouse/human CD45R/B220 antibody (clone RA3‐6B2, Biolegend Cat# 103222), Brilliant Violet 605™ anti‐mouse CD19 antibody (clone 6D5, Biolegend Cat# 115539), APC‐IgM mouse monoclonal antibody (clone II/41, ThermoFisher Cat# 17‐5790‐82), and Brilliant Violet 711™‐CD3 anti‐mouse antibody (clone 17A2, Biolegend Cat# 100241). We also used the following antibodies to detect B‐cells and LSK cells: Alexa Fluor® 700 anti‐mouse/human CD45R/B220 (clone RA3‐6B2, Biolegend, Cat# 103231), Brilliant Violet 421™ anti‐mouse CD117 (c‐kit) antibody (clone 2B8, Biolegend, Cat# 105827), CD127 (IL7R) monoclonal antibody, PE, eBioscience™ (clone A7T34, ThermoFisher Cat# 12‐1271‐82), APC anti‐mouse CD3ε antibody (clone I45‐2C11, Biolegend Cat# 100312), APC anti‐mouse TER‐119 antibody (clone TER119, Biolegend Cat# 116212), APC anti‐mouse F4/80 antibody (clone BM8, Biolegend Cat# 123116), APC anti‐mouse Nk1.1 antibody (clone PK136, Biolegend Cat# 108709), APC anti‐mouse Ly‐6G/Ly‐6C (Gr‐1) antibody (clone RB6‐8C5, Biolegend Cat# 108412), APC anti‐mouse/human CD45R/B220 antibody (clone RA3‐6B2, Biolegend Cat# 103211), APC anti‐mouse CD19 antibody (clone 6D5, Biolegend, Cat# 115511), Brilliant Violet 421™ anti‐mouse CD117 (ckit) antibody (clone 2B8, Biolegend, Cat# 105827), PE/Cy7 anti‐mouse Ly‐6A/E (Sca1) antibody (clone E13‐161.7, Biolegend Cat# 122513), CD127 (IL7R) monoclonal antibody, PE, eBioscience™ (clone A7T34, ThermoFisher Cat# 12‐1271‐82), and CD135 (Flt3) monoclonal antibody, biotin, eBioscience™ (clone A2F10, ThermoFisher Cat# 13‐1351‐81). Cells were incubated on ice for 20 min, washed twice with Flow Cytometry Staining Buffer, and resuspended in diluted SYTOXAADvanced (ThermoFisher, Cat# S10274) to exclude dead cells. Data were acquired on a BD LSRFortessa™ (BD Biosciences).

### Myeloid colony‐forming unit‐C assays

Myeloid colony‐forming unit (CFU)‐C assays were performed with MethoCult™ GF M3434 (STEMCELL Technologies Cat# 03434). Transduced GFP+ FL LSK, young BM, or FL cells were plated at 37°C 5% CO_2_ for 7–14 days before counting the colonies.

### Drug studies (monotherapy and combination therapy) in immunocompromised mice using SEM cells and primary patient sample

In total, 100,000 leukemic cells were transplanted by a tail vein injection into non‐irradiated female NSG mice; 3–4 weeks post‐transplant, once we detected human cells in the peripheral blood, we started the 12‐day treatment protocol. Day 1 is considered the first day of the treatment (first drug injection). On odd days, mice received intraperitoneal injections of acetazolamide (2 × 20 mg/kg/day), tacrolimus (1 × 10 mg/kg/day), or LB‐100 (1 × 1.5 mg/kg/day). On even days, mice were bled to monitor disease progression (NSG mice xenotransplanted with SEM cells only). On Day 12, all NSG‐SEM mice were killed for extensive post‐mortem analysis. For NSG mice xenotransplanted with cells derived from an infant KMT2A::AFF1+ BCP‐ALL patient (Edinburgh study), vehicle and acetazolamide‐treated mice were killed when a full leukemia phenotype had developed. For combination therapy in NRG‐S mice xenotransplanted with cells derived from a third infant KMT2A::AFF1+ BCP‐ALL patient (Utrecht study), 18 female immunodeficient NOD.Cg‐Rag1tm1Mom Il2rgtm1Wjl Tg(CMV‐IL3,CSF2,KITLG)1Eav/J (NRGS) mice, aged 8–12 weeks from an inhouse SP(O)F breeding colony, were transplanted intravenously with 1 × 10^6^ viable primary PDX‐derived cells lentivirally transduced to express a luciferase reporter gene. Sample size was determined a priori using a *t*‐test focused on the primary outcome parameter “survival” as determined by the continuous outcome time to event, with a standard deviation of 1.5, effect size of 2.5, an alpha of 0.05, and power of 0.8. Upon reaching ~1% human blasts in the peripheral blood, mice were randomized to the three treatment groups (*n* = 6 each) based on weight, %blasts, and the BLI signal using Randomice software.^30^ All mice received three cycles (one cycle = 1 week) of VXLC (**V**incristine 0.5 mg/kg/dose, De**x**amethasone 5 mg/kg/dose, **l** ‐asparaginase 1000 U/kg/dose, and **C**ytarabine 17.5 mg/kg/dose), with the addition of acetazolamide at 40 mg/kg/day during Cycle 1 and Cycle 2 for the VXLC + acetazolamide groups. Upon end of induction, all mice were switched from food pellets to Dietgel 93 M® and Dietgel Boost® with the addition of 400 ppm acetazolamide for the VXLC + acetazolamide + maintenance group. All drugs are administered as a single drug cocktail by an i.p. injection five times per cycle, except for vincristine, which is administered at the first day of each cycle. Tumor growth was monitored by weekly peripheral blood analyses using flow cytometry and bioluminescent imaging. For bioluminescence imaging, mice were s.c. injected with IVISbrite™ d‐Luciferin potassium salt dissolved in PBS (150 mg/kg), after which mice were anesthetized and subjected to bioluminescent imaging in a MILabs standalone Optical Imaging unit (Houten, the Netherlands). Time to event was calculated using the formula t1 + (t2 − t1)ln(Ve/V1)/ln(V2/V1), as published by Houghton et al.^31^ One animal of the VXLC + acetazolamide + maintenance group was classified as an outlier based on Grubbs's test on both BLI and peripheral blood data, and excluded from analyses. Analyses were performed in a blinded manner, based on animal number, which was grouped back to treatment group after the end of the experiment.

### Quantitative PCR for mRNA

RNA extraction and reverse transcription were carried out using the RNeasy Micro Kit (QIAGEN Cat# 74004). For reverse transcription of mRNA, we used the iScript Ready‐to‐Use cDNA Supermix of iScript Advanced cDNA synthesis kit for RT‐qPCR (Bio‐Rad Laboratories Ltd Cat# 1708841 or Cat# 1725037) according to the manufacturer's instructions. Primers were designed using Primer3 and tested. Primer sequences can be found in Supporting Information S8: Table 1. We used the Brilliant III Ultra‐Fast SYBR® Green qPCR Master Mix according to the manufacturer's instructions (Agilent Cat# 600883). Data were acquired on a QuantStudio™ 7 Flex Real‐Time PCR System (ThermoFisher). Raw data and fold change are presented in Supporting Information S14: Table 7.

### Western blot

Protein extraction was carried out in RIPA buffer (50 mM sodium chloride, 1% NP‐40, 0.5% sodium deoxycholate, 0.1% sodium dodecyl sulfate (SDS), and 50 mM Tris, pH 8.0) supplemented with a protease inhibitor cocktail tablet (Roche Cat# 04693116001). Cell pellets were washed twice in PBS, resuspended in complete RIPA buffer, and incubated on ice for 30 min. Genomic DNA was fragmented using a needle/syringe. Protein quantification was performed using the Lowry assay according to the manufacturer's instructions (BioRad Cat# 5000112). In total, 5–10 μg of total protein extract was denatured (95°C for 5 min) into 1× Laemmli buffer (Sigma Cat# S3401‐10VL) and loaded on a homemade gel (12% separation: 12% acrylamide (37.5:1), 625 mM Tris pH 8.8, 0.17% SDS, 0.08% tetramethylethylenediamine (TEMED), 0.8% ammonium persulfate (APS). 4% stacking: 4% acrylamide (37.5:1), 120 mM Tris pH 6.8, 0.1% SDS, 0.01% TEMED, 0.4% APS). Migration was carried out in Tris‐Glycine/SDS running buffer (25 mM Tris base, 190 mM glycine, and 0.1% SDS), followed by wet protein transfer to a PVDF membrane in transfer buffer (25 mM Tris base, 190 mM glycine, and 10% methanol). Membranes were blocked in 5% milk tris buffered saline with Tween® 20 (TBST) Buffer for 1 h at 4°C, with mild shaking. The primary antibody was added at a dilution of 1:1000 in 5% milk TBST Buffer and incubated overnight at 4°C, with mild shaking: human Carbonic Anhydrase VB/CA5B antibody (Biotechne Cat# AF3176), rabbit anti‐PPP2R5C antibody affinity purified (Bethyl Laboratories Inc Cat# A303‐814A), PPP3CA polyclonal antibody (Elabscience Cat# E‐AB‐14813), and monoclonal anti‐β‐actin antibody produced in mouse (Sigma Cat# A2228). Membranes were washed three times for 5 min in TBTS, followed by incubation with an horseradish peroxidase (HRP)‐conjugated secondary antibody (ThermoFisher, Cat# A16066 and A16096). Membranes were washed three times for 5 min in TBST and once for 5 min in TBS. The protein signal was detected using the Clarity™ Western ECL Substrate (BioRad Cat# 1705061) and image acquisition on the ChemiDoc Imaging System (BioRad Cat# 17001401). Image analysis and quantification were performed on Image Lab Software for PC Version 6.1 (BioRad, SOFT‐LIT‐170‐9690‐ILSPC‐V‐6‐1). Relative protein quantification for each protein was performed according to a reference band (control siRNA), and the signal for the protein of interest (CA5B, PPP3CA, or PPP2R5C) was adjusted with the relative quantification of the β‐actin signal. The β‐actin signal was acquired from the same membrane as the protein of interest. Membranes were rinsed briefly in TBS after each experiment and dried overnight at room temperature. The second staining of blot membranes was carried out 2–3 weeks later, once the signal from the previous antibody had disappeared.

### Cloning of 3′UTR into the pGL3‐promoter vector and guide RNA into the pKLV2‐U6gRNA5(BbsI)‐sEF1aBFP‐W vector

The *CA5B*, *PPP3CA*, and *PPP2R5C* UTR were amplified by PCR and cloned in the XbaI site of the pGL3‐promoter vector (Promega Cat# E1741). Guide RNAs were designed using CHOPCHOP (https://chopchop.cbu.uib.no/) and cloned into the Bbs1 site of the pKLV2‐U6gRNA5(BbsI)‐sEF1aBFP‐W vector. Oligo annealing for guide RNAs was carried out using the following method: 37°C, 30 min → 95°C, 50 min → ramp down to 25°C at 5°C/min. XbaI or Bbs1 digestion (New England Biolabs Cat# R0145S or R0539S), DNA gel extraction (QIAGEN Cat# 28704), and ligation (New England Biolabs Cat# M0202S) were conducted according to the manufacturer's instructions. Bacterial transformation was carried out in One Shot™ Stbl3™ Chemically Competent *Escherichia coli* according to the manufacturer's instructions (ThermoFisher Cat# C737303). Plasmids were purified with the QIAGEN Plasmid MiniPrep, Midiprep, or Maxiprep kits (QIAGEN Cat# 27104, 12643, and 12662).

### Luciferase assay

On the day of the transfection, HEK293T cells were harvested and seeded in DMEM 10% FCS 1%P/S 1% l‐glut at a concentration of 100,000 cells/well in a 96‐well solid white plate (Fisher Scientific Cat# 10022561). The transfection mix for each well consisted of 98 ng of the pGL3‐promoter, 100 ng of pMIRH, and 2 ng of pRL‐SV40 (Promega Cat# E2231) in 100 µL of serum‐free DMEM and 1 mg/mL of polyethyleneimine, high molecular weight (Sigma‐Aldrich Cat# 408727). The DNA solution was briefly vortexed and allowed to stand at room temperature for 15 min, before addition to a plate. Forty‐eight hours after transfection, the Firefly and Renilla luciferase activity were measured using the Dual‐Glo Luciferase Assay System (Promega Cat# E2920) and a GloMax Discover MicroPlate Reader (Promega Cat# GM3000) according to the manufacturer's instructions.

### Bioinformatic analysis of patient samples

RNA sequencing analysis of patient samples was derived from Andersson et al.'s data (EGA: EGAS00001000246).^32^ Raw gene‐level counts were normalized and variance‐stabilized using the DESeq. 2 package.^25^, ^33^ Principal component analysis (PCA) was computed using the prcomp() function in R on the transposed expression matrix containing only these three genes, without additional centering or scaling.^34^ PCA results were visualized using ggplot2,^35^ with samples colored by clinical age group (infant vs. pediatric). Exploratory associations between *CA5B*, *PPP3CA*, and *PPP2R5C* expression and *IRX1* or *HOXA9* were assessed using the Pearson correlation on variance‐stabilized expression values, with P‐values adjusted for multiple testing using the Benjamini–Hochberg false discovery rate.

### MicroRNA target prediction and data analysis

TargetScan^36^ and PicTar^37^ were used to generate a list of in silico target genes for miR‐194, miR‐99b, and miR‐125a‐5p in humans and mice. The cross‐reference of target genes between tool and species was carried out using Venny (http://bioinfogp.cnb.csic.es/tools/venny/). Graphs were generated using GraphPad Prism 6 (GraphPad Software Corporation), with statistical tests specified in the figure legends.

## RESULTS

### MiR‐194, miR‐99b, and miR‐125a‐5p are downregulated in KMT2A::AFF1+ lymphoid leukemic blasts

**Figure 1:**
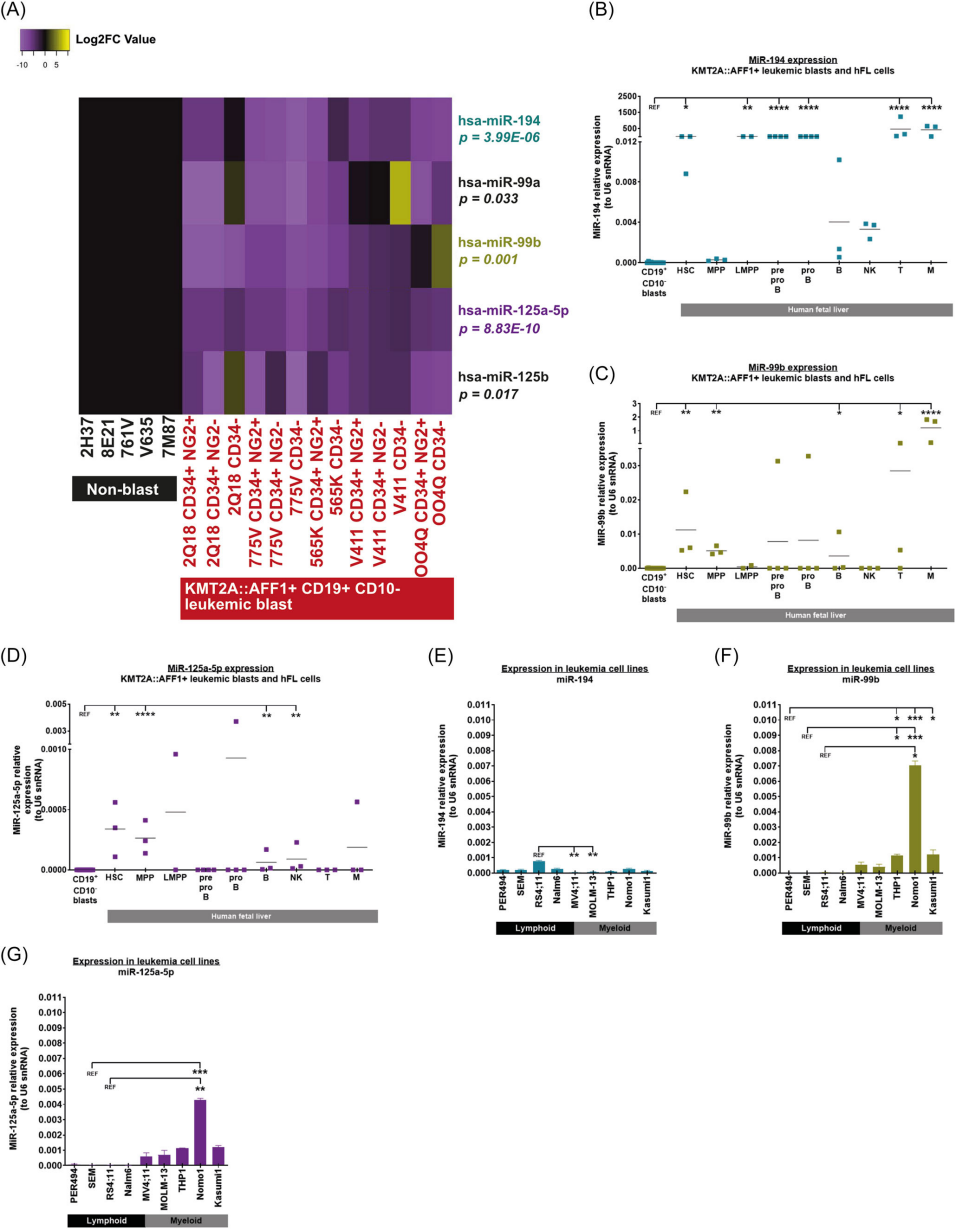
**MiR‐194, miR‐99b, and miR‐125a‐5p are all downregulated in t(4;11) KMT2A::AFF1+ B‐cell precursor acute lymphoblastic leukemia (BCP‐ALL) patients. (A)** Differential expression analysis of miR‐194, miR‐99a, miR‐99b, miR‐125a‐5p, and miR‐125b in CD19^+^CD10^−^ blasts at diagnosis (NG2^+^CD34^+^, NG2^−^CD34^+^, and CD34^−^) compared to non‐blasts at remission (CD19^−^CD10^−^). Cells were obtained from bone marrow aspirates, and data were compared using a limma test. Relative expression of **(B)** miR‐194, **(C)** miR‐99b, or **(D)** miR‐125a‐5p in KMT2A::AFF1+ leukemic blasts from KMT2A::AFF1+ BCP‐ALL patients alongside human fetal liver (FL) hematopoietic stem cell (HSC), multipotent progenitors (MPP), lymphoid‐primed multipotent progenitors (LMPP), pre‐pro‐B/pro‐B/B/NK/T lymphoid cells, and M/myeloid cells. Expression of **(E)** miR‐194, **(F)** miR‐99b, and **(G)** miR‐125a‐5p in leukemia cell lines. Data are presented as (B–D) individual data points with the mean or (E–G) mean ± SEM and compared using a Kruskall–Wallis test with a Dunn's multiple comparison test with a bilateral P‐value: P < 0.05 (*), P < 0.01 (**), P < 0.001 (***), and P < 0.0001 (****).

To identify candidate tumor suppressor genes in KMT2A::AFF1‐driven leukemogenesis, we previously performed microRNA expression profiling in infants and children with t(4;11) KMT2A::AFF1+ BCP‐ALL, which led to the identification of 85 differentially expressed microRNAs, including 19 downregulated microRNAs.^14^ This analysis compared the leukemia blasts from KMT2A::AFF1+ BCP‐ALL patients (CD19^+^CD10^−^) with the remaining BM aspirate (CD19^−^CD10^−^), but also with normal human hematopoietic stem and progenitor cells derived from the FL, CB, and adult BM. From this list, we selected three microRNA families for further analysis that showed a similar downregulation signature: miR‐194, miR‐99 family (miR‐99a and miR‐99b), and miR‐125‐5p family (miR‐125a‐5p and miR‐125b) (Figure 1A). These microRNAs were among the top 10 downregulated microRNAs.^14^ While these miRNAs have been linked to hematopoiesis and/or leukemia,^21^, ^23^, ^38^ they have not been studied in the context of KMT2A::AFF1+ BCP‐ALL. We specifically selected miR‐99b and miR‐125a‐5p because they are part of the same microRNA cluster. MiR‐194, miR‐99b, and miR‐125a‐5p are all expressed in human FL, CB, and BM, and mouse adult BM and FL HSCs and CD34^+^ cells (Figure 1B–D, Supporting Information S2: Figure 1A–F). MiR‐194 was also highly expressed in other human FL hematopoietic cells (lymphoid‐primed multipotent progenitors [LMPP], pre‐pro‐B, pro‐B, T lymphoid, and myeloid cells) compared to KMT2A::AFF1+ leukemic blasts (Figure 1B). While miR‐99b and miR‐125a‐5p were also significantly downregulated in KMT2A::AFF1+ leukemic blasts compared to human FL HSC, multipotent progenitors (MPP), and a subset of mature hematopoietic cells, their expression in human FL pre‐pro‐B and pro‐B cells was low/absent (Figure 1C,D). Interestingly, we observed low expression of miR‐194 in human FL MPP, while its expression was higher in HSC and LMPP, as well as in some differentiated hematopoietic cells (Figure 1B). This could suggest an undescribed role in HSC self‐renewal and/or lineage commitment as previously described in acute myeloid leukemia (AML) tumorigenesis.^23^

Next, we assessed the expression of miR‐194, miR‐99b, and miR‐125a‐5p in an array of leukemia cell lines. miR‐194 expression was low in all leukemia cell lines (Figure 1E). Interestingly, miR‐99b and miR‐125a‐5p expression was higher in mixed (MV4;11) and myeloid leukemia cell lines compared to lymphoid cell lines (Figure 1F,G). This correlates with previous studies highlighting their role in myeloid leukemia.^38^, ^39^, ^40^ In agreement with primary patient results, all three microRNAs also had a low/absent expression in SEM and RS4;11 KMT2A::AFF1+ BCP leukemia cell lines compared to known oncogenic co‐drivers (miR‐128a and miR‐130b) (Supporting Information S2: Figure 1G,H).^14^ Together, these results highlight the downregulation of miR‐194, miR‐99b, and miR‐125a‐5p in KMT2A::AFF1+ leukemic cells with a pro‐B phenotype.

It has previously been shown that alterations in DNA methylation are an important component of the transcriptional changes induced by KMT2A fusions.^24^, ^41^ To establish whether the downregulation of the three miRNAs in the patient samples is a consequence of DNA methylation, we analyzed their loci for differential methylation, using previously published data from KMT2A::AFF1+ patients.^24^ *MIR194‐1* (codes for miR‐194) is located in the intron of the *IARS2* gene (Supporting Information S2: Figure 1I) and, therefore, is under the same transcriptional regulation as *IARS2*.^42^ Compared with hBCPs, the upstream region around the promoter annotation of *IARS2* showed hypermethylation in KMT2A::AFF1+ patients,^24^ suggesting suppression of *IARS2* and, with it, miR‐194 (Supporting Information S2: Figure 1I). *MIR99B* and *MIR125A* are located within the same miRNA cluster inside the host gene *SPACA6* (Supporting Information S2: Figure 1I).^43^ Similar to *IARS2*, *SPACA6* also displays hypermethylation around its promoter region in KMT2A::AFF1+ patients.^24^ These results suggest that differential DNA methylation may be responsible for the downregulation of these three miRNAs in KMT2A::AFF1+ patients.^24^

### miR‐194, miR‐99b, and miR‐125a‐5p overexpression has a negative impact on the fitness of KMT2A::AFF1+ lymphoid leukemic blasts

**Figure 2:**
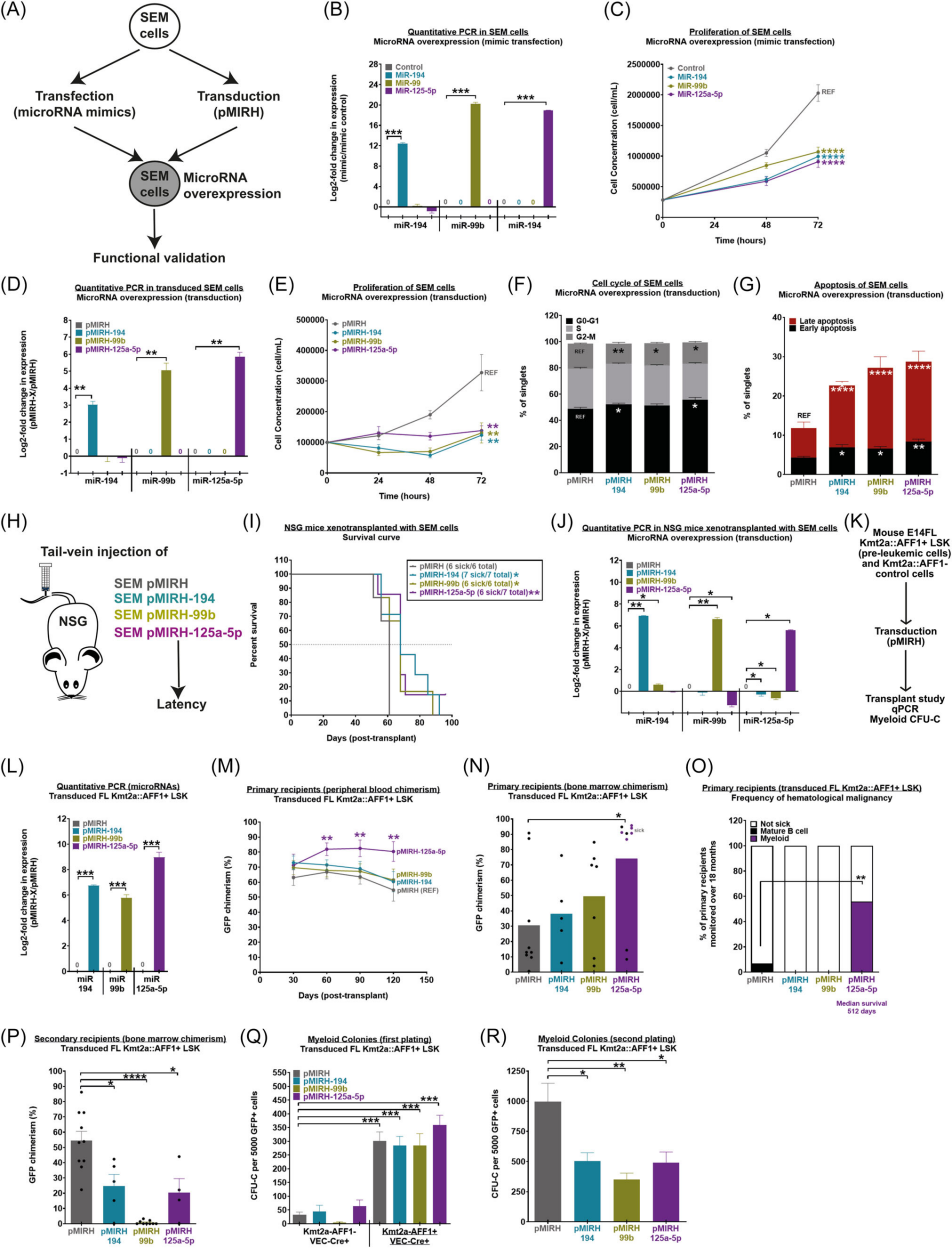
**MiR‐194, miR‐99b, and miR‐125a‐5p overexpression has a negative impact on the fitness of KMT2A::AFF1+ lymphoid leukemic blasts and fetal liver (FL) Kmt2a::AFF1+ pre‐leukemic cells. (A)** Experimental layout for functional validation in SEM cells. **(B)** Reverse transcription quantitative polymerase chain reaction (RT‐qPCR) and **(C)** proliferation of SEM cells transfected with miRVANA® mimics (*n* ≥ 3). **(D)** RT‐qPCR and **(E)** proliferation of SEM cells transduced with the pMIRH lentivirus (*n* ≥ 3). **(F)** Cell cycle analysis and **(G)** apoptosis of SEM cells overexpressing miR‐194, miR‐99b, or miR‐125a‐5p through lentiviral transduction (*n* ≥ 3). **(H)** Outline of transplantation experiments involving miRNA‐overexpressing SEM cells. **(I)** Survival curve of NSG mice xenotransplanted with SEM cells that overexpress control vector (*n* = 6), miR‐194 (*n* = 7), miR‐99b (*n* = 6), or miR‐125a‐5p (*n* = 7). Survival differences were assessed using a Gehan–Breslow–Wilcoxon test using the pMIRH group as the reference. **(J)** RT‐qPCR of the bone marrow of NSG mice xenotransplanted with SEM cells once they deceased due to KMT2A::AFF1+ leukemia. **(K)** Experimental design for the functional validation in pre‐leukemic FL Kmt2a::AFF1+ LSK and control cells. **(L)** RT‐qPCR of FL Kmt2a::AFF1+ LSK overexpressing miR‐194, miR‐99b, or miR‐125a‐5p through lentiviral transduction (*n* ≥ 3). **(M)** Peripheral blood and **(N)** bone marrow engraftment in primary recipients (Ly5.1.2) of FL Kmt2a::AFF1+ LSK that overexpress the control vector (*n* = 9), miR‐194 (*n* = 5), miR‐99b (*n* = 7), or miR‐125a‐5p (*n* = 9). **(O)** Frequency of hematological malignancies in primary recipients of FL Kmt2a::AFF1+ LSK that overexpress the control vector, miR‐194, miR‐99b, or miR‐125a‐5p. **(P)** BM chimerism of secondary recipients of FL Kmt2a::AFF1+ LSK that overexpress the control vector (*n* = 10), miR‐194 (*n* = 5), miR‐99b (*n* = 9), or miR‐125a‐5p (*n* = 4) 4 months post‐transplant. **(Q)** First plating and **(R)** second plating of FL Kmt2a::AFF1+ LSK in myeloid conditions (*n* ≥ 3). Data are presented as mean ± SEM and compared using a Mann–Whitney *U* test with a bilateral P‐value according to the indicated reference value (REF): P < 0.05 (*), P < 0.01 (**), P < 0.001 (***), and P < 0.0001 (****).

To gain a better understanding of the role of miR‐194, miR‐99b, and miR‐125a‐5p in the maintenance of KMT2A::AFF1+ leukemic cells with a pro‐B phenotype, we first conducted functional validation in SEM cells that are derived from a child with KMT2A::AFF1+ BCP‐ALL. This was done by overexpressing each microRNA individually either through the use of a microRNA mimic (transfection) or a lentiviral vector (transduction) (Figure 2A). The overexpression of each microRNA in transfected SEM cells was confirmed by RT‐qPCR (Figure 2B). SEM cells transfected with a microRNA mimic of miR‐194, miR‐99, or miR‐125 all showed reduced proliferation (Figure 2C). This phenotype was recapitulated on overexpressing each microRNA by lentiviral transduction (Figure 2D,E) and was at least partly due to changes in the cell cycle (Figure 2F). miR‐194, miR‐99b, or miR‐125a‐5p overexpression also led to increased apoptosis in SEM cells (Figure 2G). Importantly, the overexpression of miR‐194, miR‐99b, or miR‐125a‐5p in NSG mice xenotransplanted with miR‐194, miR‐99b, or miR‐125a‐5p overexpressing SEM cells prolonged the latency of KMT2A::AFF1+ BCP‐ALL (Figure 2H,I), with the overexpression of all three miRNAs maintained throughout (Figure 2J). The overexpression of miR‐194 or miR‐99b led to a small but significant decrease in *KMT2A::AFF1* expression, but the expression of its target genes (*MEIS1*, *HOXA9*, and *BCL2*) remained unchanged (Supporting Information S3: Figure 2A–D). This suggests that the KMT2A::AFF1‐regulated gene expression was stable in control and microRNA‐overexpressing cells. These results highlight a novel tumor suppressor role for miR‐194, miR‐99b, and miR‐125a‐5p in KMT2A::AFF1+ BCP‐ALL.

### miR‐194, miR‐99b, and miR‐125a‐5p decrease the self‐renewal and clonogenic potential of pre‐leukemic FL Kmt2a::AFF1+ LSK

Given the negative effect of all three microRNAs on SEM cell proliferation and survival, we also assessed their effect on the self‐renewal and clonogenic potential of pre‐leukemic FL Kmt2a::AFF1+ LSK derived from our Kmt2a::AFF1xVEC‐Cre mouse model (Figure 2K).^15^ We overexpressed the microRNAs individually in FL Kmt2a::AFF1+ LSK cells (Figure 2L) and transplanted them into Ly5.1/2 recipients to assess their role in the engraftment and self‐renewal of pre‐leukemic cells. While recipients of miR‐194+ or miR‐99b+ cells did not show significant changes in their peripheral blood chimerism compared to control recipients, miR‐125a‐5p overexpression in pre‐leukemic cells led to an increased engraftment in both the peripheral blood and BM (Figure 2M,N). Over time, this led to a subset of FL Kmt2a::AFF1+ LSK pMIRH‐125a‐5p primary recipients developing a myeloid malignancy with a long latency (512 days) (Figure 2O). Interestingly, secondary transplant experiments revealed an impaired self‐renewal potential from hematopoietic stem and progenitor cells derived from FL Kmt2a::AFF1+ LSK primary recipients that overexpress miR‐194, miR‐99b, or miR‐125a‐5p (Figure 2P). To complement these findings, we performed myeloid CFU‐C assays to assess the clonogenic potential of pre‐leukemic cells that overexpress miR‐194, miR‐99b, or miR‐125a‐5p. While clonogenic potential was unchanged in myeloid CFU‐C assays from both the FL control and Kmta2a::AFF1+ LSK upon microRNA overexpression, we observed an impaired replating activity (Figure 2Q,R) that is reminiscent of the secondary transplant results (Figure 2P). We also observed a significant decrease of *Kmt2a::AFF1* expression in FL Kmt2a::AFF1+ LSK that overexpress miR‐194, miR‐99b, or miR‐12a‐5p, which also decreased the expression of a subset of its target genes (*Meis1*, *Hoxa9*, and *Bcl2*) (Supporting Information S3: Figure 2E–H). Overall, these results reveal a role of miR‐194, miR‐99b, or miR‐125a‐5p in KMT2A::AFF1+ leukemic and pre‐leukemic cells that differs from their function in normal HSC or subtypes of AML as mentioned previously. Overall, all three microRNAs negatively affected this specific leukemia subset.

### miR‐194, miR‐99b, and miR‐125a‐5p increase the latency of Kmta2‐AFF1+ pMIRH‐128a pro‐B ALL

**Figure 3:**
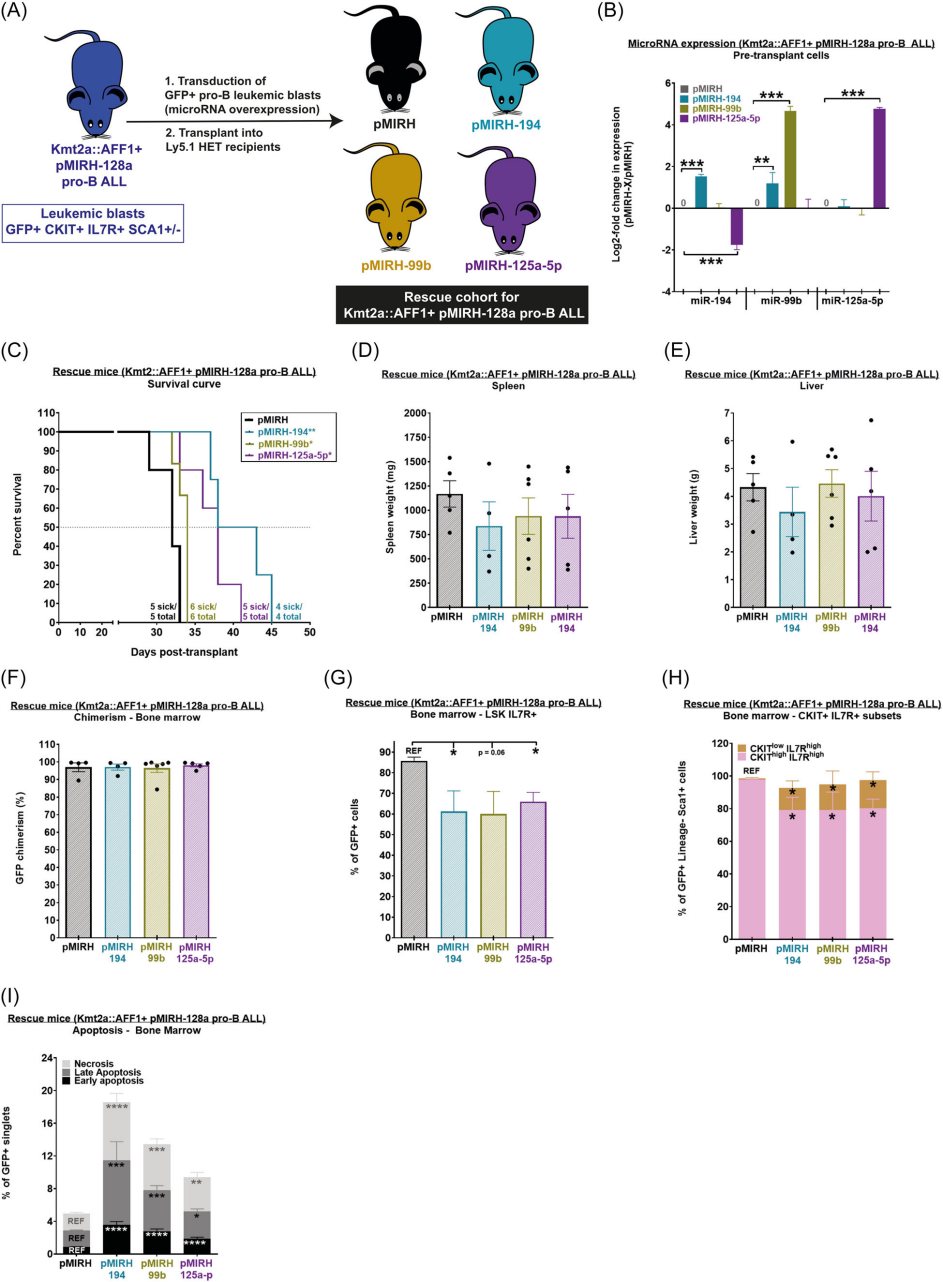
**MiR‐194, miR‐99b, and miR‐125a‐5p increase the latency of Kmt2a::AFF1+ pMIRH‐128a pro‐B acute lymphoblastic leukemia (ALL). (A)** Experimental design. MiR‐194, miR‐99b, and miR‐125a‐5p are overexpressed in GFP+ Kmt2a::AFF1+ pMIRH‐128a pro‐B leukemic blasts using a lentiviral transduction (pMIRH), followed by transplantation into Ly5.1/2 recipients. **(B)** Reverse transcription quantitative polymerase chain reaction (RT‐qPCR) in transduced Kmt2a::AFF1+ pre‐transplant cells to confirm the overexpression of miR‐194, miR‐99b, or miR‐125a‐5p (*n* ≥ 3). **(C)** Survival curve of Kmt2a::AFF1+ pMIRH‐128a mice that overexpress the control vector (*n* = 5), miR‐194 (*n* = 6), miR‐99b (*n* = 5), or miR‐125a‐5p (*n* = 4) in GFP+ Kmt2a::AFF1+ pMIRH‐128a pro‐B leukemic blasts. Survival differences were assessed using a Gehan–Breslow–Wilcoxon test. **(D)** Spleen weight, **(E)** liver weight, and **(F)** bone marrow GFP chimerism of Kmt2a::AFF1+ pMIRH‐128a B‐cell precursor (BCP)‐ALL mice that overexpress miR‐194, miR‐99b, or miR‐125a‐5p. Proportion of **(G)** LSK IL7R+ and **(H)** Lineage‐CKIT^high^IL7R+/Lineage‐CKIT^low^IL7R+ leukemic blasts in the bone marrow of Kmt2a::AFF1+ pMIRH‐128a BCP‐ALL mice that overexpress miR‐194, miR‐99b, or miR‐125a‐5p. **(I)** Apoptosis of GFP+ cells from the bone marrow of Kmt2a::AFF1+ pMIRH‐128a BCP‐ALL mice that overexpress miR‐194, miR‐99b, or miR‐125a‐5p (leukemia). Data are presented as mean ± SEM and compared using a Mann–Whitney *U* test with a bilateral P‐value according to the indicated reference value (REF): P < 0.05 (*), P < 0.01 (**), P < 0.001 (***), and P < 0.0001 (****).

Next, we assessed the impact of miR‐194, miR‐99b, and miR‐125a‐5p on the maintenance of Kmt2a::AFF1+ pMIRH‐128a BCP‐ALL, a novel syngeneic mouse model that recapitulates infant t(4;11) KMT2A::AFF1+ BCP‐ALL.^14^ This model was initially established through serial transplantation of Kmt2a::AFF1+ FL LSK pre‐leukemic cells that overexpress miR‐128a, which is a potent oncogene in KMT2A::AFF1+ infant BCP‐ALL.^14^ Similar to patients, all three microRNAs are downregulated in Kmt2a::AFF1+ pMIRH‐128a BCP‐ALL.^14^ We harvested BM GFP+ leukemic blasts of Kmt2a::AFF1+ pMIRH‐128a mice and overexpressed each microRNA individually using lentiviral transduction and transplanted them into Ly5.1/2 recipients (Figure 3A,B). Similar to NSG mice xenotransplanted with SEM cells (Figure 2I), Kmt2a::AFF1+ pMIRH‐128a BCP‐ALL mice that overexpressed miR‐194, miR‐99b, or miR‐125a‐5p showed a longer latency compared to control mice (Figure 3C). All mice eventually developed and succumbed to Kmt2a::AFF1+ pro‐B ALL (endpoint of the experiment) with hepatosplenomegaly (Figure 3D,E) and a high GFP chimerism in the BM, spleen, peripheral blood, liver, and lungs (Figure 3F, Supporting Information S3: Figure 2I–L). Interestingly, all three microRNAs led to a reduction in LSK IL7R+ leukemia‐propagating cells in the BM (Figure 3G), especially cells that were CKIT^high^ (Figure 3H, Supporting Information S3: Figure 2M). Finally, there were more apoptotic cells in Kmta2::AFF1+ pMIRH‐128a+ BCP‐ALL mice that overexpressed miR‐194, miR‐99b, and miR‐125a‐5p (Figure 3I), similar to our observation in SEM cells (Figure 2F). We also confirmed the maintained expression of all three microRNAs in Kmt2a::AFF1+ pMIRH‐128a+ rescue mice (Supporting Information S3: Figure 2N), although their expression had decreased (compare with Figure 3B). Intriguingly, this may indicate escape mechanisms and attest to the tumor suppression potential of the microRNAs. Overall, these results corroborate with our findings in SEM cells: the overexpression of miR‐194, miR‐99b, and miR‐125a‐5p impairs the maintenance of KMT2A::AFF1+ BCP‐ALL.

### miR‐194, miR‐99b, and miR‐125a‐5p act through their downstream targets CA5B, PPP3CA, and PPP2R5C

**Figure 4:**
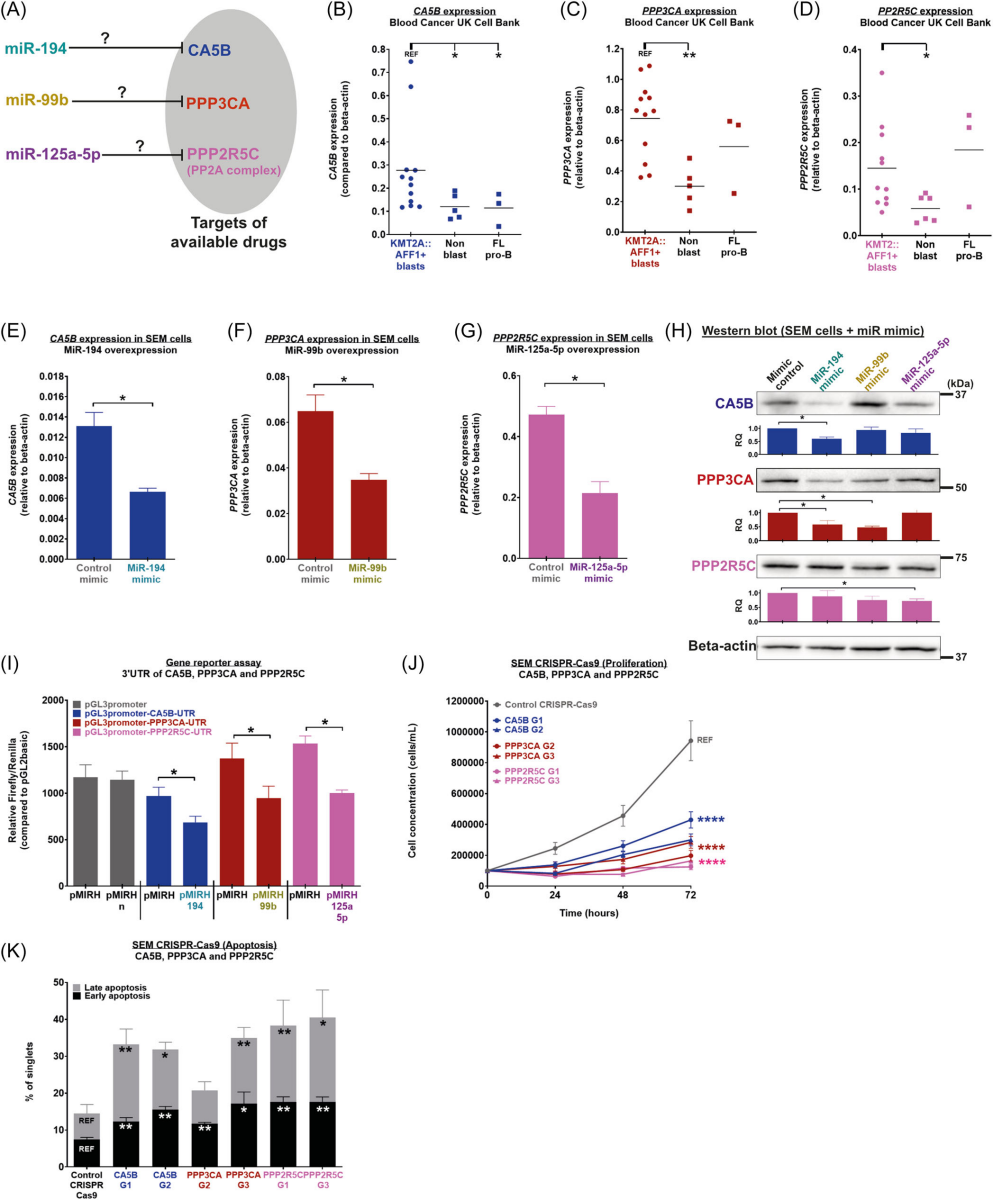
**MiR‐194, miR‐99b, and miR‐125a‐5p act through their downstream targets CA5B, PPP3CA, and PPP2R5C. (A)** CA5B, PPP3CA, and PPP2R5C are predicted targets of miR‐194, miR‐99b, and miR‐125a‐5p, respectively. **(B)** *CA5B*, **(C)** *PPP3CA,* and **(D)** *PPP2R5C* expression in leukemic blasts, non‐blasts from KMT2A::AFF1+ B‐cell precursor acute lymphoblastic leukemia (BCP‐ALL) patients, and normal FL pro‐B lymphoid cells. Reverse transcription quantitative polymerase chain reaction (RT‐qPCR) of **(E)** *CA5B*, **(F)** *PPP3CA,* and **(G)** *PPP2R5C* in SEM cells that overexpress miRVANA® mimics (*n* ≥ 3). **(H)** Western blots of SEM cells transfected with miRVANA® mimics for CA5B, PPP3CA, and PPP2R5C. The relative quantification (RQ) according to the control condition is presented. β‐Actin was used as a reference gene. **(I)** Luciferase assay in 293T cells using pGL3‐promoter‐UTR vectors of CA5B, PPP3CA, and PPP2R5C to assess microRNA‐mediated regulation (*n* ≥ 3). **(J)** Proliferation and **(K)** apoptosis of SEM CRISPR‐Cas9 cells knocked out with CA5B, PPP3CA, and PPP2R5C (*n* ≥ 3). Data are presented as mean ± SEM and compared using a Mann–Whitney *U* test with a bilateral P‐value according to the indicated reference value (REF): P < 0.05 (*), P < 0.01 (**), P < 0.001 (***), and P < 0.0001 (****).

Next, we focused on identifying the microRNA target genes downstream of miR‐194, miR‐99b, or miR‐125a‐5p that are upregulated in leukemic blasts derived from patients with KMT2A::AFF1+ BCP‐ALL. We cross‐referenced the predicted targets of all three microRNAs with patient expression data sets^44^, ^45^ and identified three genes that are upregulated in patients and that have available drugs in the clinic: CA5B (predicted target of miR‐194, inhibited by acetazolamide), PPP3CA (predicted target of miR‐99b, inhibited by tacrolimus), and PPP2R5C (predicted target of miR‐125a‐5p, part of the PP2A complex inhibited by LB‐100) (Figure 4A, Supporting Information S8: Table 1, Supporting Information S9: Table 2, and Supporting Information S10: Table 3). These drugs were selected because they are food and drug administration‐approved (acetazolamide and tacrolimus) or at advanced stages of clinical trials (LB‐100). We confirmed that these are upregulated in patients with KMT2A::AFF1+ BCP‐ALL (Figure 4B–D). *CA5B* was also upregulated in KMT2A::AFF1+ leukemic blasts compared to FL and CB pro‐B lymphoid cells (Figure 4B, Supporting Information S4: Figure 3A). *PPP3CA* and *PPP2R5C* expression in FL pro‐B lymphoid cells was similar to KMT2A::AFF1+ leukemic blasts, but was significantly higher compared to CB pro‐B lymphoid cells (Figure 4C,D, Supporting Information S4: Figure 3B,C). This could be linked to the fetal signature maintained in KMT2A::AFF1+ BCP‐ALL patients.^17^, ^18^ Importantly, these respective target genes were downregulated in Kmt2a::AFF1+ pMIRH‐128a leukemic blasts transduced with pMIRH‐194, pMIRH‐99b, and pMIRH‐125a‐5p (Supporting Information S4: Figure 3D–F).

To confirm the direct regulation of miR‐194‐CA5B, miR‐99b‐PPP3CA, and miR‐125a‐5p‐PPP2R5C, we conducted gene expression analysis in SEM cells transfected with microRNA mimics (Figure 2B). The overexpression of all three microRNAs led to significant downregulation of their respective target genes at the RNA level (RT‐qPCR) and the protein level (western blot) (Figure 4E–H). To confirm direct targeting, we cloned the 3′UTR region of each gene that is predicted to be recognized by their respective microRNAs downstream of the Firefly luciferase gene of the pGL3‐promoter vector. The significant reduction of the relative luciferase activity upon co‐transfection of each UTR with its respective microRNA confirmed the direct regulation between miR‐194‐CA5B, miR‐99b‐PPP3CA, and miR‐125a‐5p‐PPP2R5C (Figure 4I). Finally, we assessed the biological consequences of CA5B, PPP3CA, or PPP2R5C knockout in SEM cells using CRISPR‐Cas9. SEM cells were co‐transduced with a gRNA lentivirus (BFP) and Cas9 lentivirus (GFP). We confirmed the downregulation of CA5B (RNA and protein), PPP3CA (only RNA), and PPP2R5C (RNA and protein) (Supporting Information S4: Figure 3G–I). SEM cells that were knocked out with CA5B, PPP3CA, or PPP2R5C all showed a significant decrease in cell proliferation and increased cell death (Figure 4J,K). We further assessed the correlation between *CA5B*, *PPP3CA*, and *PP2R5C* expression and the mutually reciprocal *HOXA9/IRX1* expression pattern that segregates patients with disease aggressivity.^46^, ^47^, ^48^, ^49^ Exploratory analyses suggest an inverse correlation between *CA5B* and *IRX1*, and an inverse correlation between *PPP3CA* and *HOXA9* (Supporting Information S11: Table 4).^32^ While PCA based on the expression of *CA5B*, *PPP3CA*, and *PPP2R5C* did not result in complete separation of infant and pediatric samples, it revealed a partial structure along the first two principal components, suggesting heterogeneity in these transcriptional programs that may be driven by age group (Supporting Information S4: Figure 3J). Further in‐depth analyses with more samples would be needed to support these observations. Finally, STRING analysis with predicted targets of all three microRNAs that are upregulated in patients does not suggest an enrichment in interactions within this network, but does highlight an indirect interaction between PPP3CA and PPP2R5C (Supporting Information S4: Figure 3K).

These results highlight a direct link between three microRNAs that act as tumor suppressors (miR‐194, miR‐99b, and miR‐125a‐5p) and three oncogenes (CA5B, PPP3CA, and PPP2R5C) in KMT2A::AFF1+ BCP‐ALL.

### Acetazolamide, tacrolimus, and LB‐100 impair the survival of KMT2A::AFF1+ pro‐B leukemic blasts while having minimal effect on normal FL and BM mouse LSK

**Figure 5:**
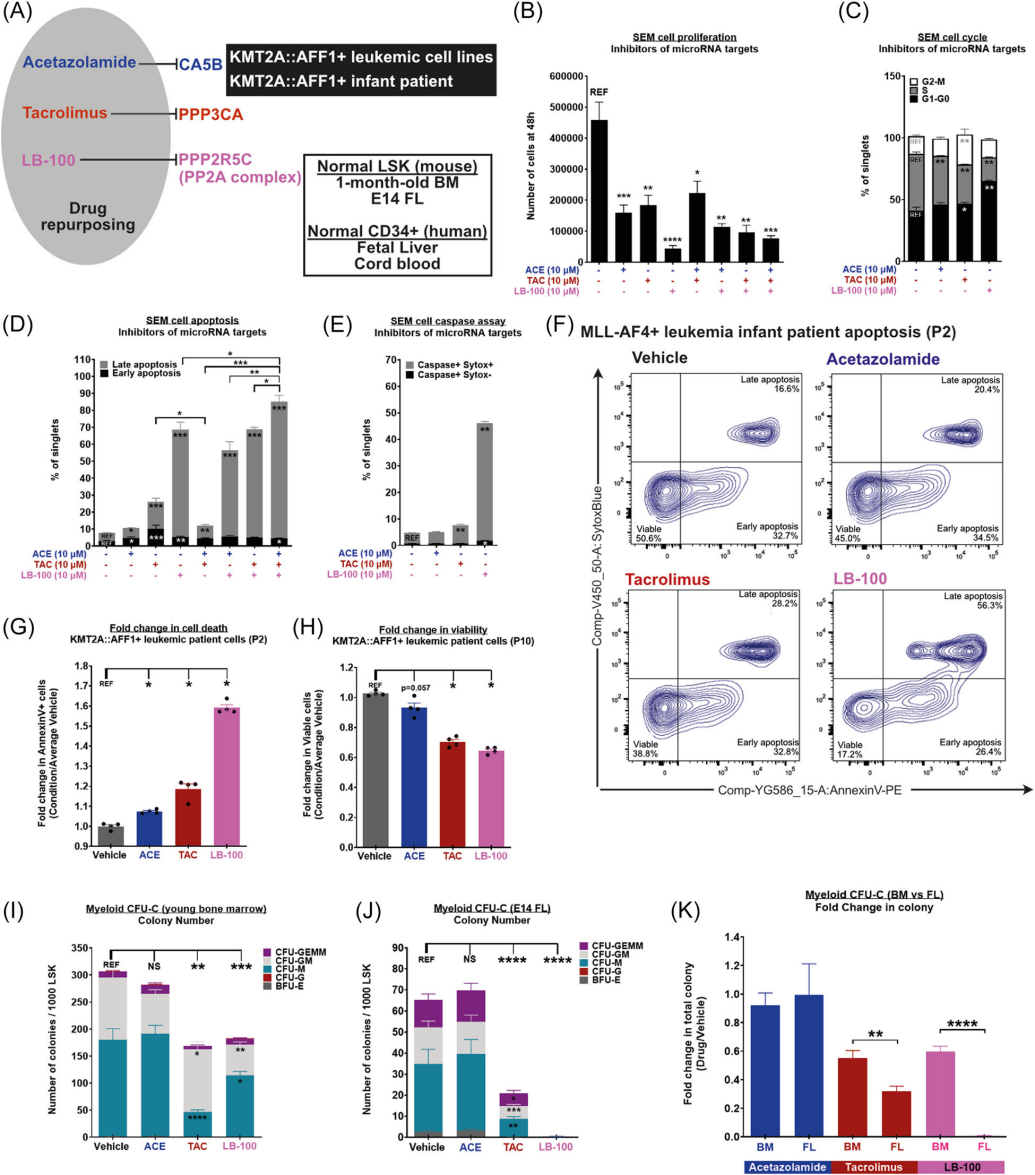
**Acetazolamide, tacrolimus, and LB‐100 impair the survival of KMT2A::AFF1+ pro‐B leukemic blasts while having minimal effect on normal fetal liver (FL) and bone marrow (BM) mouse LSK. (A)** Experimental design to assess drug effect on pro‐B leukemic cells (SEM and infant KMT2A::AFF1+ patient) and normal young BM and FL mouse LSK. **(B)** Proliferation of SEM cells exposed to acetazolamide, tacrolimus, and LB‐100 (10 μM) as single agents, or in combination (*n* ≥ 3). **(C)** Cell cycle analysis of SEM cells exposed to acetazolamide, tacrolimus, and LB‐100 (10 μM). **(D)** Apoptosis of SEM cells exposed to acetazolamide, tacrolimus, and LB‐100 (10 μM) as single agents, or in combination (*n* ≥ 3) using AnnexinV staining. **(E)** Activated caspase‐3/7 in SEM cells exposed to acetazolamide, tacrolimus, and LB‐100 (10 μM) (*n* ≥ 3). **(F)** AnnexinV apoptosis and **(G)** fold change of cell death in leukemic blasts derived from a KMT2A::AFF1+ B‐cell precursor acute lymphoblastic leukemia (BCP‐ALL) infant patient treated with acetazolamide, tacrolimus, and LB‐100 for 24 h (10 μM). **(H)** Fold change in the cell viability of leukemic blasts derived from a second KMT2A::AFF1+ BCP‐ALL infant patient treated with acetazolamide, tacrolimus, or LB‐100 (10 μM). Myeloid colony‐forming unit (CFU)‐C assay of mouse LSK derived from **(I)** young BM (1 month‐old) and **(J)** E14 FL with acetazolamide, tacrolimus, and LB‐100 (10 μM) (*n* ≥ 3). BFU‐E, primitive erythroid progenitor cells; CFU‐G, granulocyte–macrophage progenitor cells; CFU‐GEMM, multi‐potential granulocyte, erythroid, macrophage, megakaryocyte progenitor cells; CFU‐GM, granulocyte–macrophage progenitor cells; CFU‐M, macrophage progenitor cells. **(K)** Fold change in myeloid CFU‐C colony number in mouse young BM and E14 FL LSK exposed to acetazolamide, tacrolimus, and LB‐100 at 10 μM compared to vehicle (*n* ≥ 3). Data are presented as mean ± SEM and compared using a Mann–Whitney *U* test, with a bilateral P‐value according to the indicated reference value (REF): P < 0.05 (*), P < 0.01 (**), P < 0.001 (***), and P < 0.0001 (****).

Acetazolamide and tacrolimus are commonly used medications: acetazolamide for the treatment of altitude sickness and glaucoma, and tacrolimus as an immunosuppressant to prevent organ transplant rejection.^50^, ^51^ LB‐100 is currently undergoing clinical trials in small cell lung cancer and recurrent glioblastoma (NCT04560972 and NCT03027388). Acetazolamide, tacrolimus, and LB‐100 inhibit the enzymatic activity of CA5B, PPP3CA, and the PP2A complex that includes PPP2R5C, respectively (Figure 5A). We sought to determine their effect on KMT2A::AFF1+ leukemic blasts (cell lines and patient cells) and on normal mouse LSK cells derived from 1‐month‐old BM and E14 FL (Figure 5A). All leukemia cell lines tested expressed *CA5B*, *PPP3CA*, and *PPP2R5C*, albeit at varying levels (Supporting Information S5: Figure 4A–C). We first determined that 10 μM was the lowest dose at which we observed a significant effect on cell proliferation and apoptosis with the three drugs (Supporting Information S5: Figure 4D,E, respectively). Using that concentration, we exposed KMT2A::AFF1+ leukemic cell lines (SEM, RS4;11, PER‐494, and MV4;11) to all three drugs separately (10 μM), which led to a significant decrease in cell proliferation (Figure 5B, Supporting Information S5: Figure 4F–H). In SEM cells, we confirmed that this impaired cell proliferation was linked to a lower proportion of cells entering the S phase (acetazolamide, tacrolimus, and LB‐100) and the accumulation of cells in G2/M upon tacrolimus treatment, suggesting a mitotic catastrophe (Figure 5C). Furthermore, tacrolimus and LB‐100 treatment caused an accumulation of cells in G0–G1 (Figure 5C). All three drugs also led to an increase in apoptosis when incubated with SEM cells (Figure 5D), which is reminiscent of the increased apoptotic response upon miR‐194, miR‐99b, or miR‐125a‐5p overexpression (Figure 2G). Acetazolamide's effect on apoptosis was much weaker compared to tacrolimus and LB‐100, while LB‐100 seems to be the most potent drug in this context, with a combination of all three causing only slightly higher levels (Figure 5D). We also confirmed that tacrolimus and LB‐100 cell death was caspase‐dependent (Figure 5E). Importantly, primary leukemic cells derived from an infant diagnosed with t(4;11) KMT2A::AFF1+ BCP‐ALL displayed a similar apoptotic response to acetazolamide, tacrolimus, and LB‐100 (Figure 5F,G). Leukemic blasts derived from a second infant diagnosed with t(4;11) KMT2A::AFF1+ BCP‐ALL were also sensitive to acetazolamide (P = 0.057), tacrolimus, and LB‐100 (Figure 5H).

Next, we exposed normal mouse LSK cells derived from young BM (1 month old to mimic the age of patients at treatment) and E14 FL to all three drugs and conducted myeloid colony‐forming assays to determine their effect on hematopoietic clonogenic potential. Upon the first plating, acetazolamide did not alter the colony‐forming capacity of young BM and E14 FL mouse LSK cells (Figure 5I,J). Tacrolimus and LB‐100 both led to a significant decrease in hematopoietic colony‐forming ability in both young BM and E14 FL mouse LSK cells (Figure 5I,J), but this effect was significantly stronger in E14 FL LSK (Figure 5K). Overall, these results show that acetazolamide, tacrolimus, and LB‐100 can negatively affect the proliferation and survival of KMT2A::AFF1+ leukemic blasts in vitro, while they display less toxicity on normal young LSK compared to FL LSK. Preliminary results using human FL and human CB CD34^+^ cells also suggest that acetazolamide and LB‐100 are more toxic to hFL CD34^+^ cells compared to human CB, while tacrolimus was toxic to both (Supporting Information S5: Figure 4I,J). These features (strong anti‐leukemic activity and moderate toxicity toward normal LSK in vitro) make these drugs attractive candidates for the treatment of infants and children with t(4;11) KMT2A::AFF1+ BCP‐ALL.

### Acetazolamide, tacrolimus, and LB‐100 decrease the leukemia burden in KMT2A::AFF1+ BCP‐ALL mice

**Figure 6:**
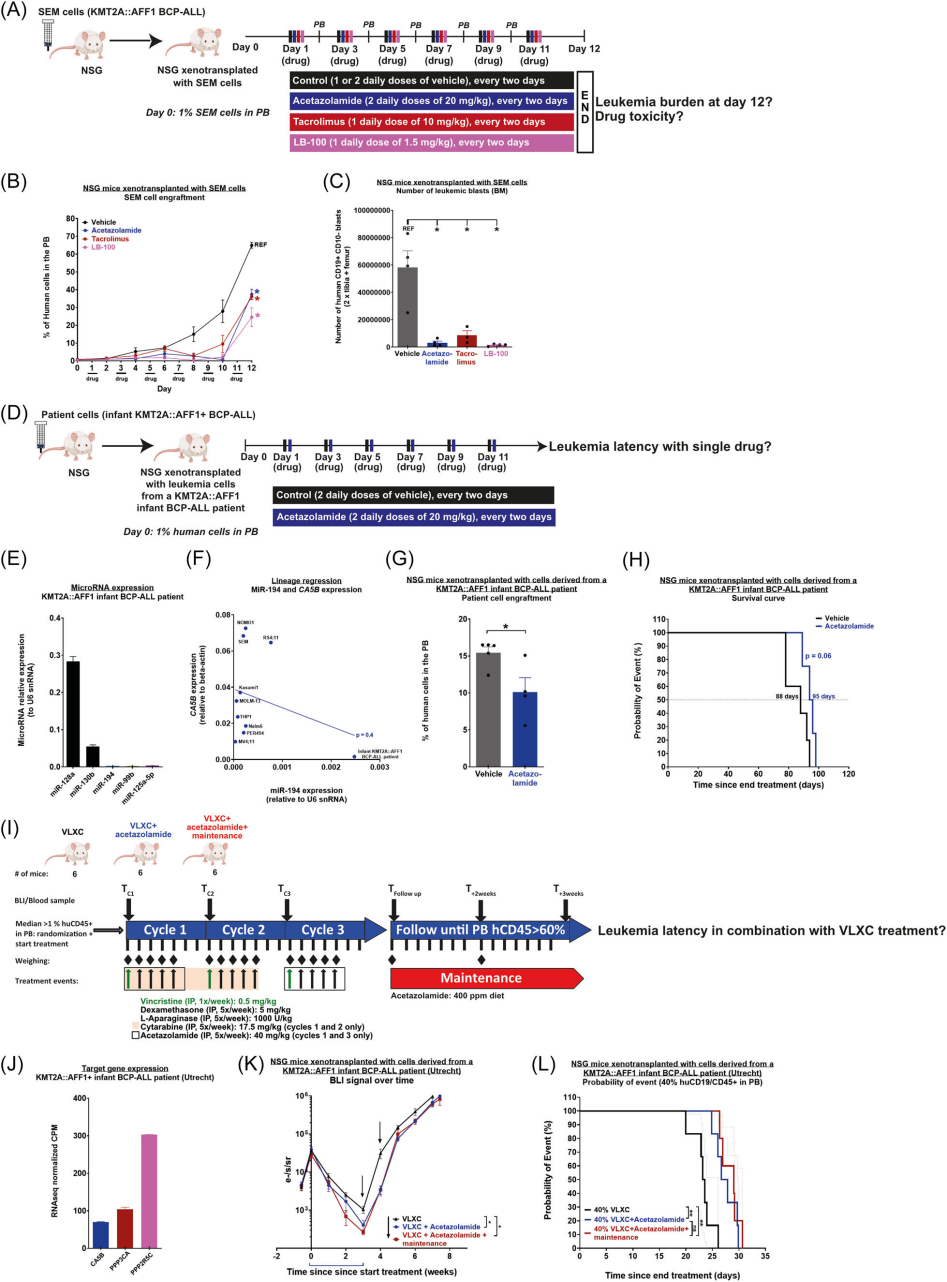
**Acetazolamide, tacrolimus, and LB‐100 impair the maintenance of KMT2A::AFF1+ B‐cell precursor acute lymphoblastic leukemia (BCP‐ALL). (A)** Experimental design of the drug study to assess the effect of acetazolamide, tacrolimus, and LB‐100 in the maintenance of KMT2A::AFF1+ BCP‐ALL in NSG mice xenotransplanted with SEM cells. The treatment was initiated when 1% human cells were detected in the peripheral blood of NSG‐SEM mice. **(B)** Human cell contribution in the peripheral blood over the course of the 12‐day treatment. **(C)** Absolute number of SEM cells in the bone marrow of NSG mice (vehicle and drug) at Day 12. **(D)** Experimental design of the monotherapy drug study to assess the effect of acetazolamide on the survival of NSG mice xenotransplanted with cells derived from an infant KMT2A::AFF1+ BCP‐ALL patient. **(E)** Reverse transcription quantitative polymerase chain reaction (RT‐qPCR) of miR‐128a, miR‐130b, miR‐194, miR‐99b, and miR‐125a‐5p in leukemic cells from the infant KMT2A::AFF1+ BCP‐ALL patients used in (D). **(F)** Correlation of CA5B and miR‐194 expression in leukemic cell lines and the primary sample used in (D). **(G)** Human cell contribution in the peripheral blood 5 days after the end of the treatment. **(H)** Survival curve of NSG mice xenotransplanted with cells derived from an infant KMT2A::AFF1+ BCP‐ALL patient: vehicle‐ and acetazolamide‐treated. **(I)** Experimental design of combination therapy to assess the effect of acetazolamide alongside an induction therapy on the survival of NRG‐S PDX mice xenotransplanted with cells derived from an infant KMT2A::AFF1+ BCP‐ALL patient. **(J)** CA5B, PPP3CA, and PPP2R5C expression in an infant KMT2A::AFF1+ BCP‐ALL patient (Utrecht). **(K)** Total body luminescence signal (BLI) over time during and after the treatment for VXLC (*n* = 6), VXLC + acetazolamide (*n* = 6), and VXLC + acetazolamide + maintenance (*n* = 5) groups. Data are presented as mean ± SEM and compared using a Welch's *t*‐test. **(L)** Survival curve of VXLC‐, VXLC + acetazolamide‐, and VXLC + acetazolamide + maintenance‐treated groups. Survival differences were assessed using a Gehan–Breslow–Wilcoxon test. Data are presented as mean ± 95% CI. P < 0.05 (*), P < 0.01 (**), P < 0.001 (***), and P < 0.0001 (****).

Given that acetazolamide, tacrolimus, and LB‐100 severely impaired the survival of KMT2A::AFF1+ leukemic blasts in vitro, we next evaluated if they could decrease leukemia burden in vivo. For the first drug study, we used immunocompromised NSG mice xenotransplanted with the BCP‐ALL cell line SEM to assess the overall toxicity and efficacy of each separate drug against KMT2A::AFF1+ BCP‐ALL. Once human leukemic cells were detected in the peripheral blood (~1%), we initiated a 12‐day treatment period (Figure 6A). All mice were killed the day after the last drug injection to assess the overall leukemia burden in the peripheral blood, BM, spleen, and liver. First, during the course of the treatment, we observed a reduced percentage of KMT2A::AFF1+ leukemic blasts in the peripheral blood of all drug‐treated mice (Figure 6B). This effect became noticeable at Day 8, which is when the leukemia burden of untreated/vehicle mice increased exponentially. Notably, half of the vehicle mice developed full‐blown leukemia already at Day 9 of treatment and had to be killed before the endpoint (Supporting Information S6: Figure 5A). This reduced percentage of KMT2A::AFF1+ leukemic blasts in the peripheral blood of all drug‐treated mice was maintained at Day 12 (Figure 6B). Vehicle mice showed a significant decrease in their body weight over the course of treatment, most likely a result of leukemia development (Supporting Information S6: Figure 5A). Acetazolamide‐treated mice showed no significant weight loss, while Tacrolimus‐ and LB‐100‐treated mice showed significant weight loss before the exponential phase of KMT2A::AFF1+ BCP‐ALL (Supporting Information S6: Figure 5B–D). This suggests that tacrolimus and LB‐100 are more toxic to mice compared to acetazolamide.

Next, we quantified KMT2A::AFF1+ leukemia burden in the BM, spleen, and liver of all mice at Day 12 (the day after the last injection). We also included the vehicle mice that developed full‐blown leukemia at Day 9. Strikingly, we observed 6–10 times fewer human CD19^+^CD10^−^ leukemic cells in the BM of all drug‐treated mice (Figure 6C). This trend was also observed in the spleen of acetazolamide‐treated mice; these had smaller spleens compared to vehicle mice, which correlates with the reduced leukemic infiltration observed in the BM (Supporting Information S6: Figure 5E). The liver size was similar between vehicle and treated mice (Supporting Information S6: Figure 5F). This suggests that the drug sensitivity of KMT2A::AFF1+ leukemic cells could be variable between tissues, perhaps due to differences in tissue drug distribution.

Given that acetazolamide showed the most promising anti‐leukemia activity and the lowest toxicity among all three drugs, we decided to assess its efficacy on the survival of NSG mice xenotransplanted with primary patient‐derived infant KMT2A::AFF1+ BCP‐ALL cells (Figure 6D). This patient displayed the microRNA expression signature that we observed in KMT2A::AFF1+ BCP‐ALL patients (high miR‐128a and miR‐130b, low miR‐194, miR‐99b, and miR‐125‐5p) (Figure 6E).^14^ This patient expressed *CA5B* at a lower level compared to KMT2A::AFF1+ leukemic cell lines, which was also true for *PPP3CA* and *PPP2R5C* (Figure 6F, Supporting Information S6: Figure 5G,H). However, the expression level of the target gene correlated with their respective microRNAs. Acetazolamide significantly reduced the human leukemia burden in the peripheral blood of these PDX mice (Figure 6G). PDX mice treated with acetazolamide also survived for a longer period (P = 0.06) compared to vehicle‐treated mice (88 days vs. 95 days) after treatment completion (Figure 6H). Given that the treatment was short and administered approximately 80 days before the development of full‐blown leukemia, these results suggest that acetazolamide has anti‐leukemic activity.

To further verify the anti‐leukemia activity of acetazolamide and to establish whether it could be combined with current chemotherapy protocols, we administered acetazolamide together with an induction therapy regimen (VXLC: vincristine, dexamethasone, l‐asparaginase, and cytarabine) mimicking that of infant KMT2A::AFF1+ BCP‐ALL patients (Figure 6I). Immunocompromised NRG‐S mice were transplanted with KMT2A::AFF1+ leukemic blasts derived from an additional infant patient diagnosed with KMT2A::AFF1+ BCP‐ALL. RNAseq analysis performed on the leukemic blasts of this patient confirmed the expression of *CA5B*, *PPP3CA,* and *PPP2R5C* (Figure 6J). Prior to transplantation, these human leukemia cells were made to stably express a luciferase gene to allow disease monitoring by bioluminescence (BLI). Once ~1% engraftment was reached in the peripheral blood, mice were randomized into three treatment groups: VXLC (induction only, three cycles), VXLC + acetazolamide (induction with acetazolamide at Cycles 1 and 3), and VXLC + acetazolamide + maintenance (induction with acetazolamide at Cycles 1 and 3, followed by acetazolamide maintenance through their diet). Although the initial aim was to provide acetazolamide treatment for the complete induction period, given that no signs of toxicity were observed during monotreatment, the protocol was adopted as the combination of acetazolamide with complete induction appeared to be too toxic. Instead, acetazolamide was provided during the first cycle to provide a very heavy treatment hit, followed by a second cycle of normal induction, after which acetazolamide could be safely added during the third treatment cycle when cytarabine is no longer administered. Strikingly, the addition of acetazolamide to the induction therapy led to a significantly deeper remission compared to induction treatment alone, as measured by total body luminescence upon completion of the induction therapy regimen (Figure 6K). However, the addition of acetazolamide was not enough to completely eradicate the disease, as the BLI signal starts rising again after treatment halt. A similar pattern was also observed at the peripheral blood level, although it did not reach significance due to the limit of detection of the flow cytometer (Supporting Information S7: Figure 6A). Interestingly, the signal observed in the peripheral blood appeared to lag 1 week behind the total body BLI signal, suggesting that peripheral blood as a proxy does display the response in the niches, although with a delay. The addition of acetazolamide to the induction therapy also significantly prolonged the survival of KMT2A::AFF1+ mice (Figure 6L). Interestingly, we also observed a trend toward prolonged survival in KMT2A::AFF1+ acetazolamide‐treated mice that were subsequently placed on acetazolamide maintenance (the VXLC + acetazolamide + maintenance group) (Supporting Information S7: Figure 6B). While this trend was not significant, acetazolamide maintenance through the diet was very tolerable and a higher dose might indeed significantly prolong survival. Hence, using acetazolamide as maintenance therapy for KMT2A::AFF1+ patients could prove beneficial in the long term, given its low toxicity.

Overall, this study has identified three drugs that could be beneficial for the treatment of infant KMT2A::AFF1+ BCP‐ALL patients.

## DISCUSSION

The aim of this study was to identify novel therapeutic avenues for KMT2A::AFF1+ BCP‐ALL patients using their unique microRNA expression signature. Given that microRNAs are negative regulators of gene expression, we selected three microRNAs that are downregulated in infant KMT2A::AFF1+ BCP‐ALL patients: miR‐194, miR‐99b, and miR‐125a‐5p.^14^ miR‐194 can act as a tumor suppressor in myeloid leukemic cells,^23^ while the miR‐99b/let7e/miR‐125a‐5p cluster is important for maintaining the HSC pool in human and mice.^21^, ^52^, ^53^ miR‐99 and miR‐125 families can also drive skewing toward the myeloid lineage and contribute to the development of myeloid malignancies. It is worth mentioning that miR‐125a‐5p overexpression in FL KMT2A::AFF1+ LSK pre‐leukemic cells caused myeloid malignancy in about 50% of recipients, which suggests that it can promote myeloid potential in KMT2A::AFF1+ hematopoietic stem and progenitor cells. Notably, miR‐99 family members are usually downregulated in ALL patients compared to AML patients.^54^ Therefore, we decided to investigate if the downregulation of miR‐194, miR‐99b, and miR‐125a‐5p in infant KMT2A::AFF1+ BCP‐ALL was linked to an unknown tumor‐suppressive role in this specific leukemia subtype.

We conducted extensive functional validation in vitro and in vivo using a variety of mouse and human models of infant and pediatric KMT2A::AFF1+ BCP‐ALL to strengthen our conclusions. First, we overexpressed all three microRNAs separately in SEM leukemic cells that are derived from a child with KMT2A::AFF1+ BCP‐ALL. Their overexpression decreased proliferation and increased apoptosis in SEM cells. All three microRNAs also prolonged the survival of KMT2A::AFF1+ BCP‐ALL mice (NSG mice xenotransplanted with SEM and KMT2A::AFF1+ pMIRH‐128a pro‐B ALL). In addition, we observed decreased CKIT expression upon miR‐194, miR‐99b, or miR‐125a‐5p overexpression in KMT2A::AFF1+ pMIRH‐128a pro‐B ALL, suggesting partial release of the lineage differentiation arrest at the pro‐B stage. These results suggest that microRNA function can be similar between leukemia subtypes (miR‐194),^23^ but can also differ significantly (miR‐99b and miR‐125a‐5p).^38^, ^39^

We next aimed to identify relevant target genes for each microRNA that could be treated by novel drugs that are readily available for clinical use, thereby fast‐tracking a potential translational route. We focused on CA5B (miR‐194 target), PPP3CA (miR‐99b target), and PPP2R5C (miR‐125a‐5p target), which can be inhibited by acetazolamide, tacrolimus, and LB‐100, respectively. CA5B is a mitochondrial carbonic anhydrase that catalyzes the reversible hydration of carbon dioxide, which is essential to many biological functions, including pH balance, cell metabolism, and enzymatic activity.^55^ PPP3CA is a protein phosphatase that responds to calcium signaling to activate specific gene expression programs,^56^ while the PP2A phosphatase complex, which comprises the PPP2R5C subunit, is a major signaling hub in both normal and malignant cells.^57^ All three genes are overexpressed in KMT2A::AFF1+ BCP‐ALL patients and are direct targets of their respective microRNAs. The inhibition of CA5B, PPP3CA, or PPP2R5C with acetazolamide, tacrolimus, or LB‐100 recapitulated the phenotype observed upon their respective microRNA overexpression. Notably, acetazolamide showed very little toxicity toward normal mouse young BM and E14 FL LSK cells. Tacrolimus and LB‐100 showed moderate toxicity toward normal LSK cells, but to a lesser extent than toward KMT2A::AFF1+ leukemic cells. Their effect also appeared to be stronger in FL LSK cells, which have a more proliferative phenotype and a gene expression signature similar to infant KMT2A::AFF1+ BCP‐ALL patients and are considered to be potential cells of origin.^17^, ^18^ It would be important to also test the effect of the drugs on normal human haematopoietic cells. Our preliminary data suggest no toxicity on human FL or CB cells, although further data points are needed, including from human BM cells. Overall, these results show a very promising response of KMT2A::AFF1+ leukemic cells to acetazolamide, tacrolimus, and LB‐100. They also highlight novel pathways that can influence the leukemia microenvironment and cell metabolism (CA5B), but also intracellular signaling and gene expression (PPP3CA and PPP2R5C). In fact, acetazolamide is currently in a Phase I clinical trial for MGMT Promoter‐Methylated IDH Wildtype Glioblastoma (NCT03011671).

Finally, we assessed the efficiency of acetazolamide, tacrolimus, and LB‐100 in reducing the leukemia burden in two mouse models of KMT2A::AFF1+ BCP‐ALL: NSG mice xenotransplanted with SEM cells or cells derived from an infant KMT2A::AFF1+ BCP‐ALL patient. Strikingly, each drug was individually able to reduce the leukemia burden in the peripheral blood and BM of NSG mice xenotransplanted with SEM cells. Furthermore, acetazolamide as a single agent prolonged the survival of NSG mice xenotransplanted with cells derived from an infant KMT2A::AFF1+ BCP‐ALL patient. Given that acetazolamide displayed no/mild toxicity toward normal hematopoietic cells and there were no side effects on mice, this drug could become a valuable addition to the therapeutic regimen for infants with KMT2A::AFF1+ BCP‐ALL or could be used as maintenance therapy. Most importantly, a combination therapy regimen (induction+acetazolamide) was efficient at significantly reducing the leukemia burden, resulting in a deeper remission, and prolonging the survival of KMT2A::AFF1+ NSG mice xenotransplanted with cells derived from an infant KMT2A::AFF1+ BCP‐ALL patient, even when only administered during part of the induction treatment regimen. The introduction of cytarabine in 1999 led to improvement in the prognosis, but this drug induces toxicities in patients. Notably, treatment‐related mortalities due to chemotherapy toxicity are as frequent as death from relapsed/refractory disease. Therefore, finding a drug that is less toxic and more specific has become one of the main clinical goals for infant KMT2A::AFF1+ BCP‐ALL patients. This study paves the way toward exploring the use of acetazolamide as an alternative to cytarabine for infant KMT2A::AFF1+ BCP‐ALL patients, either through complete or partial replacement, to reduce the need for this very toxic chemotherapy drug. It would also be important to consider how acetazolamide could be used in conjunction with immunotherapies, such as blinatumomab. While blinatumomab has been proven to be a powerful treatment for infant KMT2A::AFF1+ BCP‐ALL patients, severe complications such as cytokine release syndrome can arise quickly during the treatment and become life‐threatening.^58^, ^59^, ^60^ Cases of non‐responders and therapy evasion (e.g., through antigen downregulation) have also been reported, and blinatumomab is less effective in treating central nervous system disease. Therefore, having a toolbox of complementary therapeutic approaches for an aggressive and rapidly evolving cancer such as infant KMT2A::AFF1+ BCP‐ALL patients is essential. This study also identified two additional drugs that were detrimental to the survival of KMT2A::AFF1+ leukemic cells and disease maintenance in vivo: tacrolimus and LB‐100. However, in contrast to acetazolamide, tacrolimus and LB‐100 showed high toxicity in vivo when used as single drugs. Therefore, we did not combine them with VLXC chemotherapy in vivo, but they may still be considered for maintenance therapy, something to be explored in future work. In our study, acetazolamide showed the most promising addition to the chemotherapy regimen of KMT2A::AFF1+ BCP‐ALL patients with high anti‐leukemia activity and low toxicity. This has the potential to improve the treatment for a class of patients that has seen little improvement over the past 20 years.

## AUTHOR CONTRIBUTIONS

**Camille Malouf**: Conceptualization; investigation; funding acquisition; writing—original draft; methodology; validation; visualization; writing—review and editing; formal analysis. **Alasdair Duguid**: Investigation; writing—review and editing; methodology; visualization. **Kirsten S. Vrenken**: Writing—review and editing; visualization; methodology; investigation. **Tom Leah**: Investigation. **Ragini Medhi**: Investigation; methodology; visualization; writing—review and editing. **Giuseppina Camiolo**: Investigation. **Leslie Nitsche**: Investigation; writing—review and editing. **Hélène Jakobczyk**: Investigation. **Rishi S. Kotecha**: Writing—review and editing; resources. **Richard A. Anderson**: Writing—review and editing; resources. **Neil A. Barrett**: Resources. **Owen P. Smith**: Resources. **Ronald W. Stam**: Writing—review and editing; resources. **Katrin Ottersbach**: Conceptualization; writing—original draft; project administration; supervision; resources; funding acquisition; formal analysis; validation.

## CONFLICT OF INTEREST STATEMENT

The authors declare no conflicts of interest.

## FUNDING

This study was supported by grants from the Kay Kendall Leukaemia Fund (KKL871) and Cancer Research UK (C57303/A23581 and DRCNPG‐May23/100002; K.O.), the Dutch Cancer Society (R.W.S), and by the Fight Kids Cancer Funding Programme, supported by Imagine For Margo, Fondation KickCancer, Foundatioun Kriibskrank Kanner, Federazione Italiana Associazioni Genitori e Guariti Oncoematologia Pediatrica, and Cris Cancer Foundation (FKC‐Cure2MLL) (R.W.S. and K.O.). The Ottersbach laboratory is also part of the MRC National Mouse Genetic Network Haem cluster (MC_PC_21043; https://nmgn.mrc.ukri.org/clusters/haem/), which funds the generation of infant leukemia mouse models in the laboratory. This study was supported by Blood Cancer UK (19005).

## Supporting information

Supporting Information.

Supporting Information.

Supporting Information.

Supporting Information.

Supporting Information.

Supporting Information.

Supporting Information.

Supporting Information.

Supporting Information.

Supporting Information.

Supporting Information.

Supporting Information.

Supporting Information.

Supporting Information.

## Data Availability

All the data can be found in the figures of the article or from the referenced papers. No large data sets were generated that would require depositing in a data repository.
