# Supplementary material for: A microRNA expression signature in infant t(4;11) KMT2A::AFF1+ BCP‐ALL uncovers novel therapeutic targets
Source: Hemasphere. 2026 Apr 23;10(4):e70353. doi: 10.1002/hem3.70353 (PMC13103725; doi:10.1002/hem3.70353)
Supplement: Supplementary file 1 — Supporting Information. [file HEM3-10-e70353-s011.docx]

**Supplemental material for “A microRNA expression signature in infant t(4;11) KMT2A::AFF1 BCP-ALL uncovers novel therapeutic targets”**

- Supplemental Figure Legends

**Supplemental Figure Legends**

**Supplemental Figure 1.** **MiR-194, miR-99b and miR-125a-5p are all downregulated in t(4;11) KMT2A::AFF1+ BCP-ALL patients.** (A) MiR-194, (B) miR-99b and (C) miR125a-5p RT-qPCR in patient KMT2A::AFF1+ CD19+ CD10- leukemic blasts, CD34+ human cord blood and CD34+ human bone marrow. (D) MiR-194, (E) miR-99b and (F) miR-125a-5p expression in normal mouse FL and adult bone marrow hematopoietic stem cells (Lineage- CD150+ CD48- EPCR+), hematopoietic progenitors (ckit+CD34+) and differentiated hematopoietic cells (CD45+CD34-). MIR-128a, miR-130b, miR-194, miR-99b and miR-125a-5p expression in (G) SEM and (H) RS4;11 leukemia cell lines. (I) Methylation profile of the promoters regulating miR-193 (*IARS2* host gene) and the miR-99b/let-7e/miR-125a-5p cluster (*SPACA6* host gene). Data are presented as Mean ± SEM and compared using a Mann-Whitney U test with bilateral p-value: p < 0.05 (*), p < 0.01 (**), p < 0.001 (***) and p < 0.0001 (****).

**Supplemental Figure 2. MiR-194, miR-99b and miR-125a-5p impair Kmt2a::AFF1+ leukemia maintenance.** (A) *KMT2A::AFF1*, (B) *MEIS1*, (C) *HOXA9* and (D) *BCL2* RT-qPCR in SEM cells that overexpress pMIRH (control vector), pMIRH-194, pMIRH-99b or pMIRH-125a-5p. (E) *Kmt2a::AFF1*, (F) *Meis1*, (G) *Hoxa9* and (H) *Bcl2* RT-qPCR in FL Kmt2a::AFF1+ LSK that overexpress pMIRH (control vector), pMIRH-194, pMIRH-99b or pMIRH-125a-5p. GFP chimerism in the (I) spleen, (J) peripheral blood, (K) liver and (L) lungs of Kmt2a::AFF1+ pMIRH-128a BCP-ALL mice that overexpress control (n=5), miR-194 (n=6), miR-99b (n=5) or miR-125a-5p (n=4). (M) Proportion of CKIT^high^IL7R+ and CKIT^low^IL7R+ leukemic blasts (Lineage- Sca1+ fraction) in the spleen of Kmt2a::AFF1+ pMIRH-128a BCP-ALL mice that overexpress miR-194, miR-99b or miR-125a-5p. (N) RT-qPCR to confirm the overexpression of miR-194, miR-99b or miR-125a-5p in the bone marrow of rescue mice (end of experiment/leukemia) (n ≥ 3). Data are presented as Mean ± SEM and compared using a Mann-Whitney U test with bilateral p-value according to the indicated reference value (REF): p < 0.05 (*), p < 0.01 (**), p < 0.001 (***) and p < 0.0001 (****).

**Supplemental Figure 3. CA5B, PPP3CA and PPP2R5C explain the impaired KMT2A::AFF1+ leukemia maintenance observed upon miR-194, miR-99b and miR-125a-5p overexpression, respectively.**  (A) *CA5B*, (B) *PPP3CA* and (C) *PPP2R5C* expression in KMT2A::AFF1+ BCP-ALL infant patient and cord blood B-cell progenitors (CB BCP) (GSE79450). (D) *Ca5b*, (E) *Ppp3ca* and (F) *Ppp2r5c* expression in KMT2A::AFF1+ pMIRH-128a pre-transplant cells (GFP+ pro-B cells transduced with pMIRH, pMIRH-194, pMIRH-99b or pMIRH-125a-5p) (n ≥ 3). (G) *CA5B*, (H) *PPP3CA* and (I) *PPP2R5C* expression in SEM cells transduced with Cas9-GFP and KPL474-guideRNA-BFP. RT-qPCR (top) and western blot (low) are shown (n ≥ 3). (J) Principal component analysis (PCA) was performed using variance-stabilized (VST) expression values of *CA5B*, *PPP3CA*, and *PPP2R5C* across all samples. Each point represents an individual patient sample and is colored according to clinical group (infant, blue; pediatric, orange). (K) STRING analysis of the upregulated target genes for miR-194, miR-99b and miR-125a-5p to identify known and predicted protein interactions. Data are presented as Mean ± SEM and compared using a Mann-Whitney U test with bilateral p-value according to the indicated reference value (REF): p < 0.05 (*), p < 0.01 (**), p < 0.001 (***) and p < 0.0001 (****).

**Supplemental Figure 4. Acetazolamide, Tacrolimus and LB-100 impair the survival of KMT2A::AFF1+ pro-B leukemic blasts while having minimal effect on normal FL and BM mouse LSK.** (A) *CA5B*, (B) *PPP3CA* and (C) *PPP2R5C* expression by RT-qPCR of PER494, SEM, RS4;11 and MV4;11 leukemia cell lines. (D) Proliferation at 48h (0h=100 000 cells) and (E) apoptosis of SEM cells exposed to the combination of acetazolamide, tacrolimus and LB-100 (n ≥ 3). Proliferation of (F) PER494, (G) RS4;11 and (H) MV4;11 leukemia cells exposed to Acetazolamide, Tacrolimus or LB-100 (10 μM) (n ≥ 3). Apoptosis (AnnexinV+ cells) of (I) CD34+ hFL cells (n=2) or (J) CD34+ hCB cells (n=1) exposed to acetazolamide, tacrolimus or LB-100 (10 μM). Data are presented as Mean ± SEM and compared using a Mann-Whitney U test with bilateral p-value according to the indicated reference value (REF): p < 0.05 (*), p < 0.01 (**), p < 0.001 (***) and p < 0.0001 (****).

**Supplemental Figure 5.** **Acetazolamide, Tacrolimus and LB-100 impair the maintenance of KMT2A::AFF1+ pro-B ALL.** Weight fluctuation of NSG mice xenotransplanted with SEM cells over the course of the drug treatment: (A) Vehicle (n=4), (B) Acetazolamide (n=4), (C) Tacrolimus (n=4) and (D) LB-100 (n=4). (E) Spleen and (F) liver sizes of vehicle and drug-treated NSG mice xenotransplanted with SEM cells. Correlation of expression between (G) *PPP3CA* and miR-99b and (H) *PPP2R5C* and miR-125a-5p using linear regression. (I) Spleen and (J) liver sizes of NSG mice xenotransplanted with cells derived from an infant KMT2A::AFF1+ BCP-ALL patient when treated with vehicle or Acetazolamide. Data are presented as Mean ± SEM and compared using a Mann-Whitney U test with bilateral p-value: p < 0.05 (*), p < 0.01 (**), p < 0.001 (***) and p < 0.0001 (****).

**Supplemental Figure 6. Combination therapy of PDX-KMT2A::AFF1+ BCP-ALL mice.** (A) Human peripheral blood during and after the combination therapies for all three groups of mice: VXLC, VXLC+acetazolamide and VXLC+acetazolamide+maintenance groups. (B) Survival benefit displayed as time to event (TTE) of VLXC, VLXC+acetazolamide and VLXC+acetazolamide+maintenance groups, with events defined as reaching 1, 5, 10, 25 or 40% of human blasts in the peripheral blood. All datapoints are presented as box whiskers and compared using a Welch’s t-test: p < 0.05 (*), p < 0.01 (**), p < 0.001 (***) and p < 0.0001 (****).
