# Supplementary figures and images for: A microRNA expression signature in infant t(4;11) KMT2A::AFF1+ BCP‐ALL uncovers novel therapeutic targets
Source: Hemasphere. 2026 Apr 23;10(4):e70353. doi: 10.1002/hem3.70353 (PMC13103725; doi:10.1002/hem3.70353)

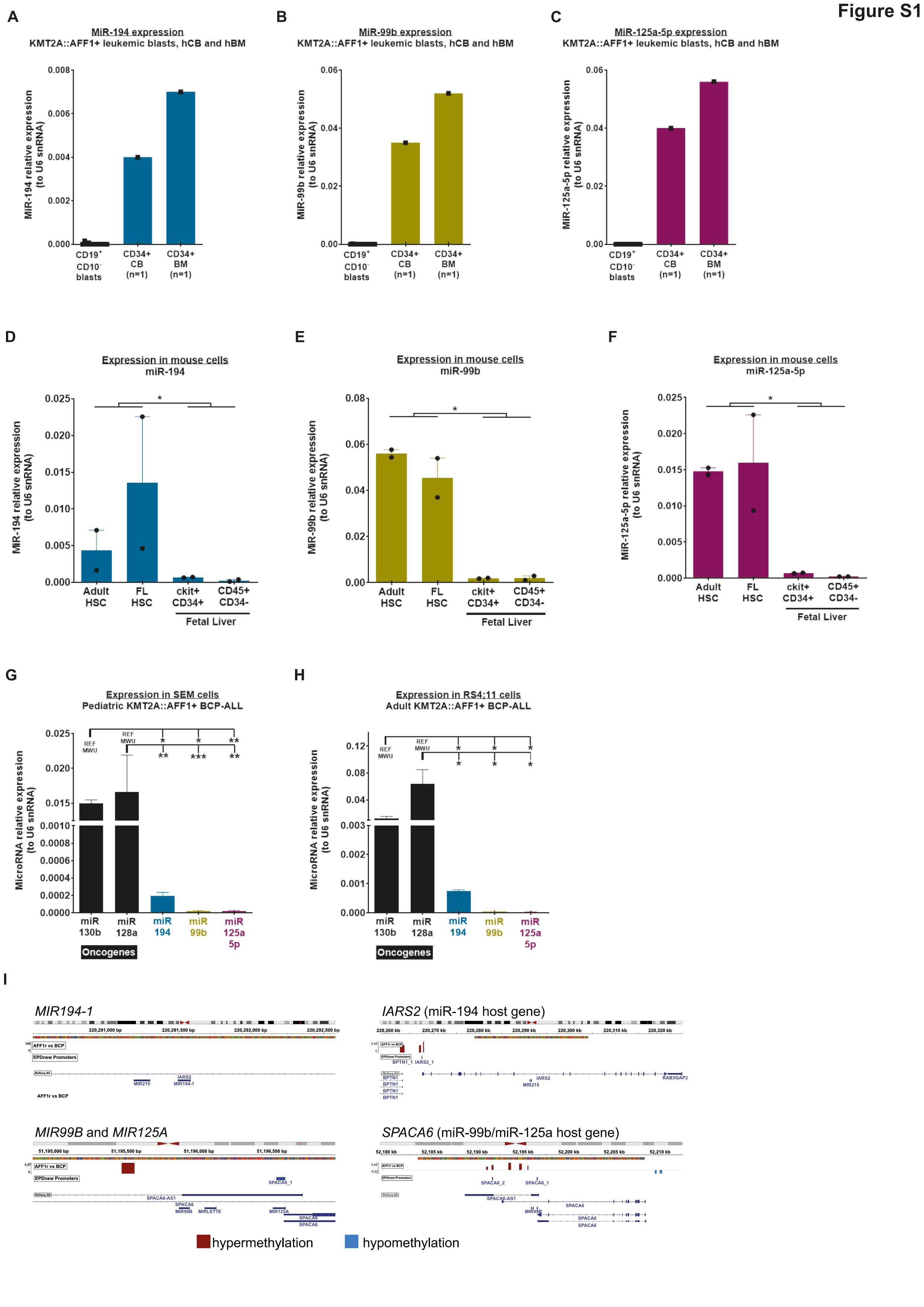

Supplement: Supplementary file 2 — Supporting Information. [file HEM3-10-e70353-s001.tiff]

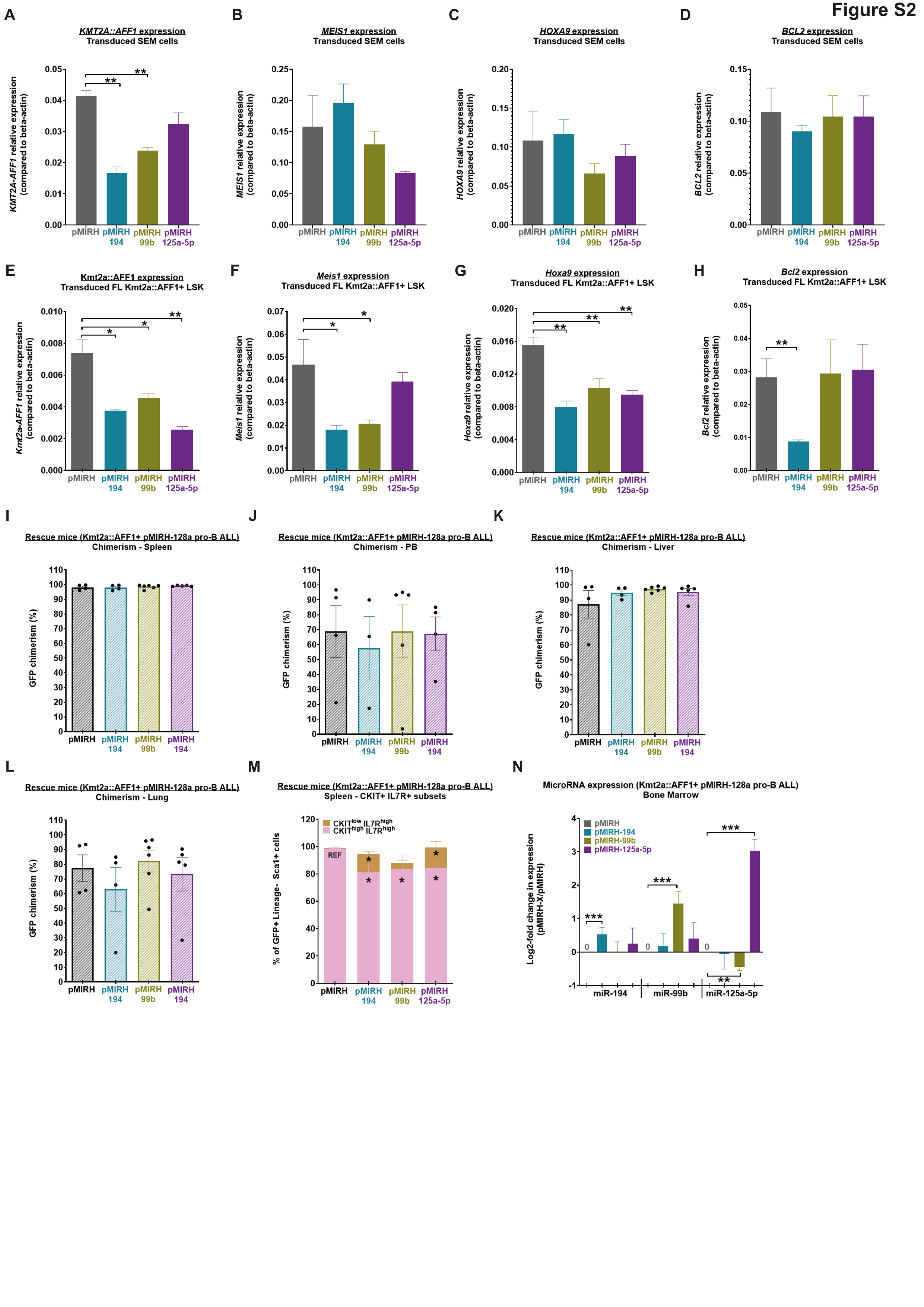

Supplement: Supplementary file 3 — Supporting Information. [file HEM3-10-e70353-s006.tiff]

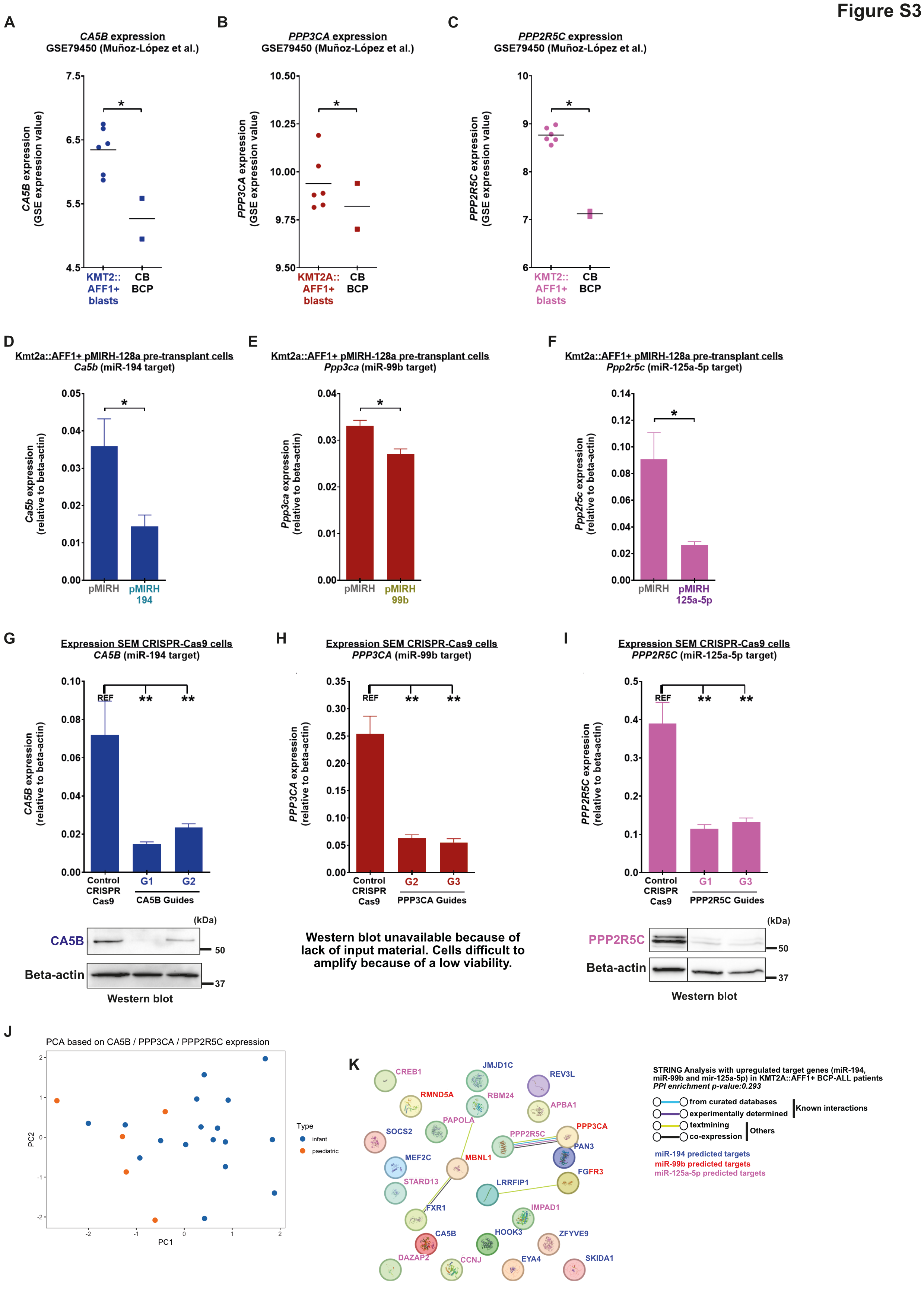

Supplement: Supplementary file 4 — Supporting Information. [file HEM3-10-e70353-s014.tiff]

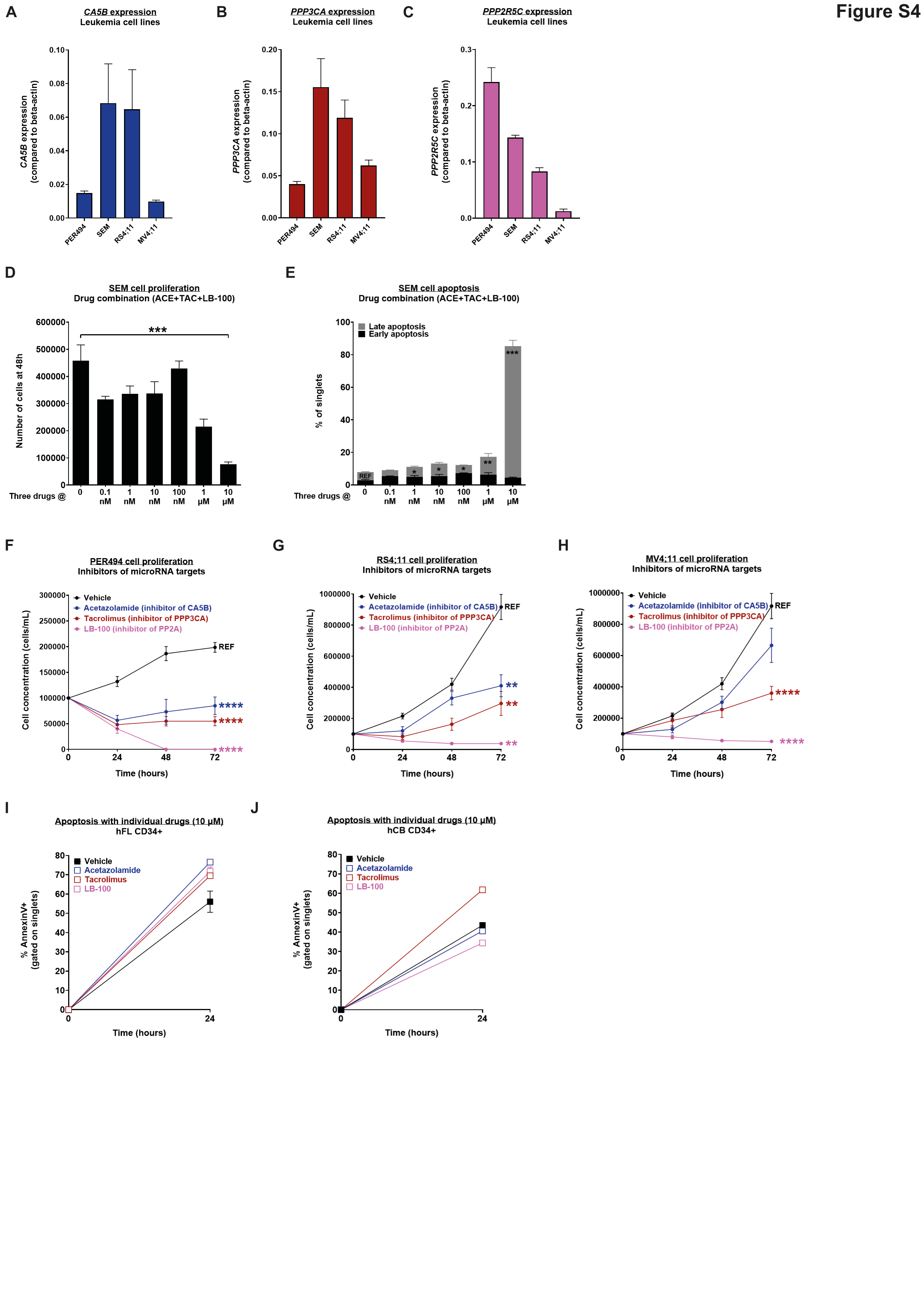

Supplement: Supplementary file 5 — Supporting Information. [file HEM3-10-e70353-s004.tiff]

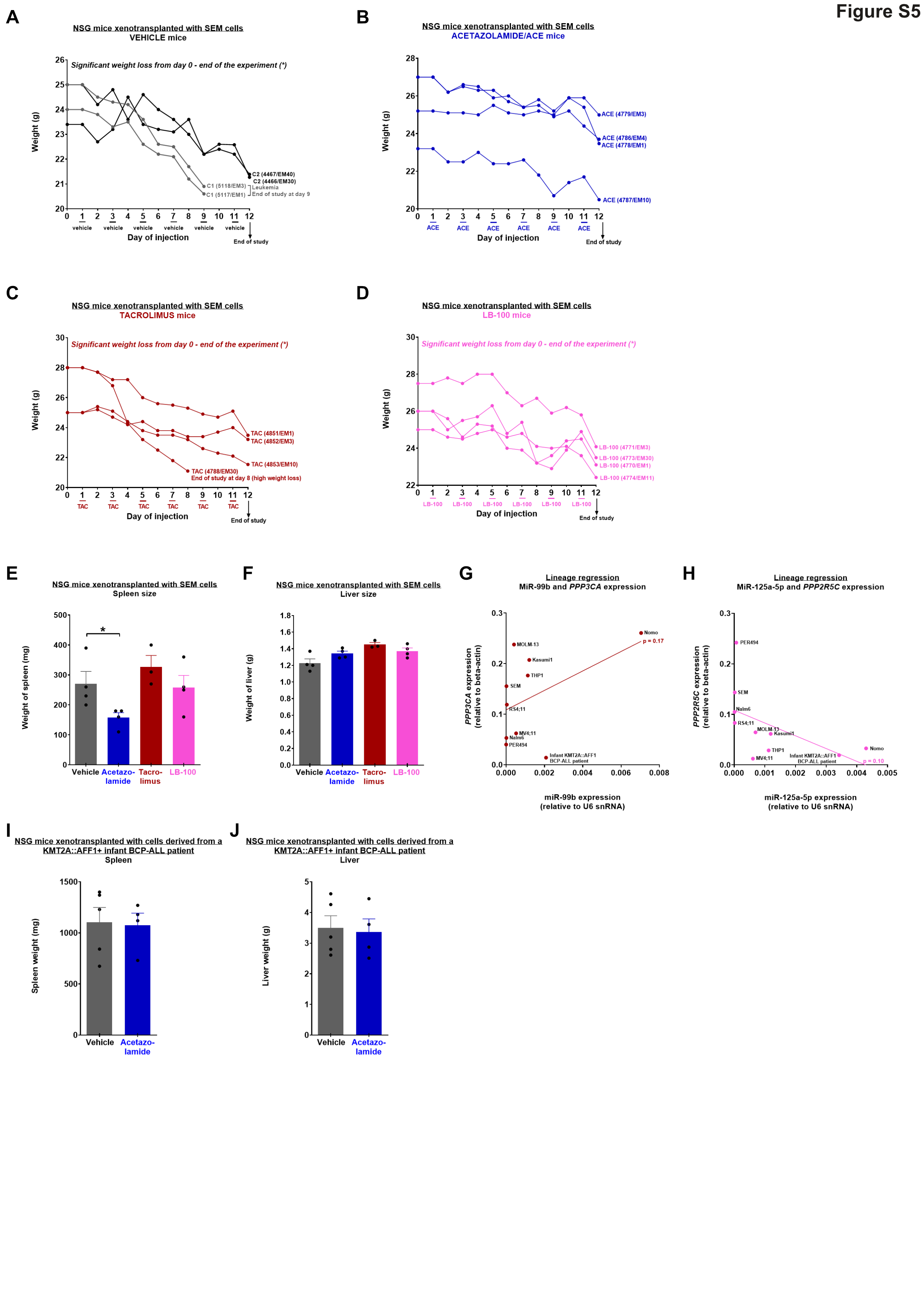

Supplement: Supplementary file 6 — Supporting Information. [file HEM3-10-e70353-s010.tiff]

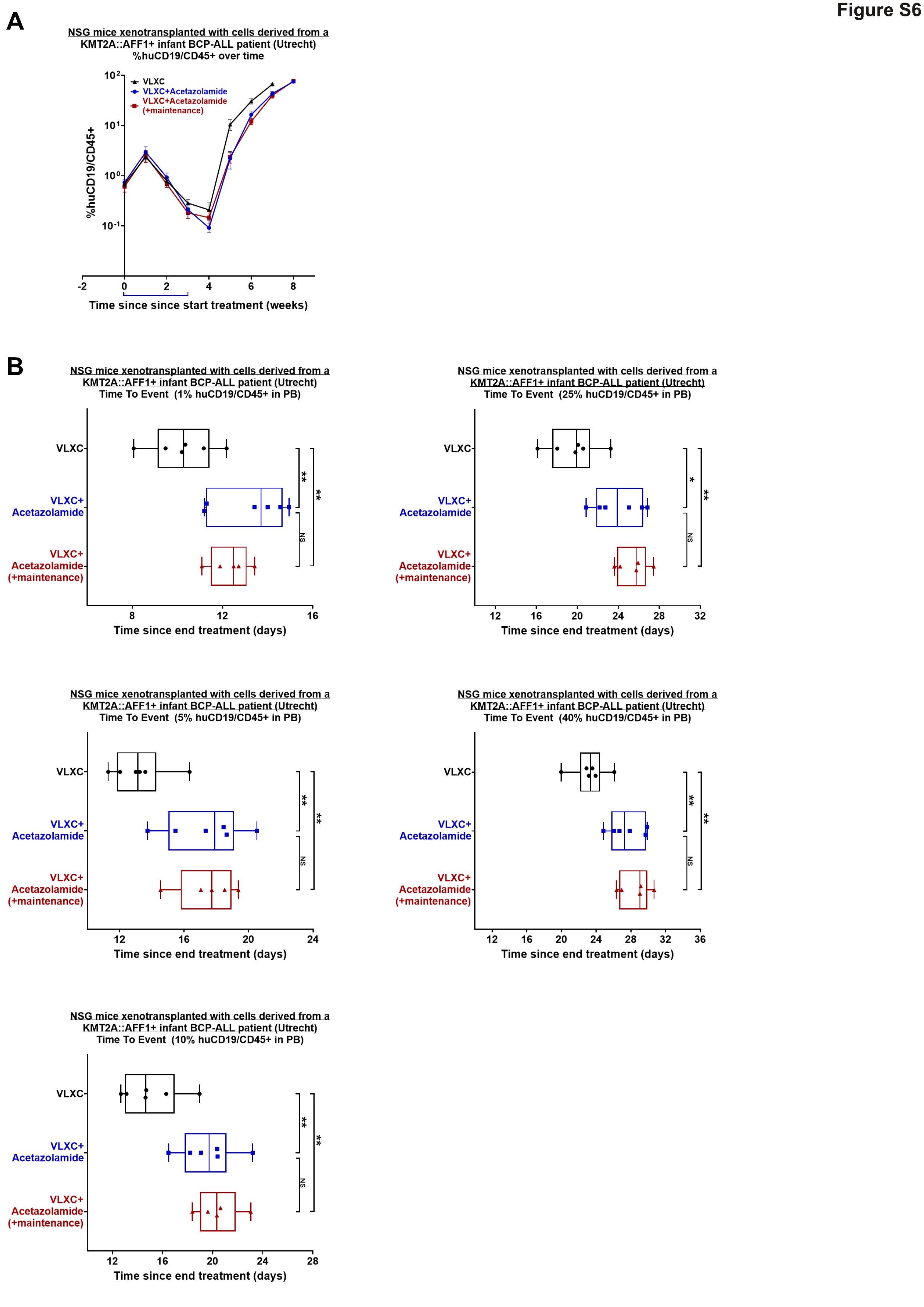

Supplement: Supplementary file 7 — Supporting Information. [file HEM3-10-e70353-s009.tiff]
